# Delivering nutrition interventions to women and children in conflict settings: a systematic review

**DOI:** 10.1136/bmjgh-2020-004897

**Published:** 2021-04-08

**Authors:** Shailja Shah, Zahra Ali Padhani, Daina Als, Mariella Munyuzangabo, Michelle F Gaffey, Wardah Ahmed, Fahad J Siddiqui, Sarah Meteke, Mahdis Kamali, Reena P Jain, Amruta Radhakrishnan, Anushka Ataullahjan, Jai K Das, Zulfiqar A Bhutta

**Affiliations:** 1 Centre for Global Child Health, Hospital for Sick Children, Toronto, Ontario, Canada; 2 Division of Women and Child Health, Aga Khan University, Karachi, Pakistan; 3 Health Services and Systems Research, Duke-NUS Graduate Medical School, Singapore

**Keywords:** nutrition, child health

## Abstract

**Background:**

Low/middle-income countries (LMICs) face triple burden of malnutrition associated with infectious diseases, and non-communicable diseases. This review aims to synthesise the available data on the delivery, coverage, and effectiveness of the nutrition programmes for conflict affected women and children living in LMICs.

**Methods:**

We searched MEDLINE, Embase, CINAHL, and PsycINFO databases and grey literature using terms related to conflict, population, and nutrition. We searched studies on women and children receiving nutrition-specific interventions during or within five years of a conflict in LMICs. We extracted information on population, intervention, and delivery characteristics, as well as delivery barriers and facilitators. Data on intervention coverage and effectiveness were tabulated, but no meta-analysis was conducted.

**Results:**

Ninety-one pubblications met our inclusion criteria. Nearly half of the publications (n=43) included population of sub-Saharan Africa (n=31) followed by Middle East and North African region. Most publications (n=58) reported on interventions targeting children under 5 years of age, and pregnant and lactating women (n=27). General food distribution (n=34), micronutrient supplementation (n=27) and nutrition assessment (n=26) were the most frequently reported interventions, with most reporting on intervention delivery to refugee populations in camp settings (n=63) and using community-based approaches. Only eight studies reported on coverage and effectiveness of intervention. Key delivery facilitators included community advocacy and social mobilisation, effective monitoring and the integration of nutrition, and other sectoral interventions and services, and barriers included insufficient resources, nutritional commodity shortages, security concerns, poor reporting, limited cooperation, and difficulty accessing and following-up of beneficiaries.

**Discussion:**

Despite the focus on nutrition in conflict settings, our review highlights important information gaps. Moreover, there is very little information on coverage or effectiveness of nutrition interventions; more rigorous evaluation of effectiveness and delivery approaches is needed, including outside of camps and for preventive as well as curative nutrition interventions.

**PROSPERO registration number:**

CRD42019125221.

**Key questions**

**What is already known?**

- Women and children affected by conflict suffer from high burden of malnutrition and poor nutritional outcomes.
- In recent decades, conflict related deaths resulting from malnutrition and other health problems have increased significantly to the extent where they are now being considered a public health problem.

**What are the new findings?**

- General food distribution was the most common intervention provided in the included studies.
- Most of the intervention were delivered to refugee populations in camp settings and through community-based approaches.
- Very few studies reported on coverage and effectiveness of the nutritional interventions.
- Key delivery facilitators included community advocacy and social mobilisation, effective monitoring and the integration of nutrition and other sectoral interventions and services.
- Key delivery barriers included insufficient resources, nutritional commodity shortages, security concerns, poor outcome reporting, limited cooperation and difficulty accessing and following-up of beneficiaries.

**What do the new findings imply?**

- Studies and research should be conducted to generate and strengthen evidence on coverage, access and improvement in delivery of nutritional interventions in context to conflict setting with rigorous and improved assessment methods.
- Studies should also study on multi-sectoral programming approach and its integration with early childhood development and mental health, which is an emerging issue.

## Introduction

Armed conflict is defined as ‘a political conflict in which armed combat involves the armed forces of at least one state (or one or more armed factions seeking to gain control of all or part of the state), and in which people have been killed by the fighting during the course of the conflict’.^1^ It originates due to social, individual and cultural differences, which leads to poverty, violence, malnutrition and mortality.^2^

Globally, 136 million people are in need of assistance due to conflict, while 52 million children suffer from acute malnutrition where disease epidemics are a global threat.^3 4^ Low/middle-income countries (LMICs) face triple burden of malnutrition,^5^ which is more complicated where there is increase in protracted and recidivist conflict, population displacement and urban warfare.^6^

According to a recent systematic review by Blanchet *et al*,^7^ several nutritional programmes have been implemented by humanitarian organisations to aid vulnerable populations during emergency settings.^8^ According to recent Sphere guidance, although food insecurity is one cause of malnutrition, providing food assistance to vulnerable population is unlikely to contribute to a long lasting solution.^9^ Thus, a multi-sectoral approach to food and nutrition response in conflict settings has been advocated. Successful nutrition interventions in non-conflict nutrition settings are characterised by a combination of political commitment, multi-sectoral collaboration, community engagement, community-based service delivery platform, and wider programme coverage and compliance.^10^ In conflict settings, consensus on specific guidelines uptake is still obscure, hence, stronger scientific evidence of implementing effective nutrition interventions is required.

In this review we aimed to synthesise the data and information currently available on how nutrition interventions for women and children have been delivered in conflict settings, and the reported barriers and facilitators of programme delivery. We also aimed to synthesise the available data on the coverage of nutrition programmes for women and children in such settings and their effectiveness.

## Methods

This systematic review on nutritional interventions is a part of series of reviews in conflict settings which includes delivery of mental health, sexual and reproductive health and other interventions in conflict settings.^11–18^ This review adheres to the Preferred Reporting Items for Systematic Reviews and Meta-Analyses statement (online supplemental appendix 1),^19^ and its protocol is registered with PROSPERO (the international prospective register of systematic reviews, www.crd.york.ac.uk/prospero/).

10.1136/bmjgh-2020-004897.supp1 Supplementary data

### Search strategy and selection criteria

We systematically searched MEDLINE, Embase, CINAHL and PsycINFO online databases for indexed journal articles published between 1 January 1990 and 31 March 2018 using search terms relating to women, children or adolescents accessing or receiving nutrition-specific interventions in conflict or post-conflict settings in LMICs (online supplemental appendix 2). In addition to the indexed literature, we also searched grey literature published between 1 January 2013 and 30 November 2018 on the websites of 10 major humanitarian organisations who are actively involved in responding to or researching conflict situations: Emergency Nutrition Network, International Committee of the Red Cross, International Rescue Committee, Médecins Sans Frontières, Save the Children, United Nations Population Fund, United Nations High Commissioner for Refugees, UNICEF, Women’s Refugee Commission and World Vision. We used broad terms for conflict and health interventions tailored to the search functionality of each website.

We deduplicated all retrieved indexed records using Endnote X7 software,^20^ and then imported unique records into Covidence software,^21^ where two reviewers independently conducted screening of each title and/or abstract for relevance. Discrepancies between reviewers’ decisions were resolved via discussion, or by a third reviewer if necessary. A single reviewer then assessed the full text of each potentially relevant publication to determine eligibility for the review. An eligible publication needed to describe a nutrition-specific intervention being delivered during or within five years of cessation of armed conflict to neonates, children, adolescents or women of reproductive age. Same approach was used for assessing grey literature.

For both indexed and grey literature, we excluded case reports of single patients; studies reporting on military personnel, refugee populations in high-income countries, surgical techniques or economic or mathematical modelling; editorials and opinion pieces; guidelines; and reviews.

### Data extraction

We extracted relevant qualitative and quantitative information from eligible publications using a structured, pilot tested data abstraction tool in REDCap software in duplicate.^22^ Discrepancies between reviewers’ data were resolved via discussion, or by a third reviewer if necessary.

### Data analysis and synthesis

We descriptively analysed the key characteristics of the included publications, focal populations and reported interventions, including their delivery characteristics, and we tabulated reported estimates of intervention coverage and effectiveness. We compared reported nutrition treatment intervention coverage and effectiveness estimates with key indicators outlined in the *Sphere Handbook*^9^ of minimum standards in humanitarian response. We narratively synthesised information on delivery barriers and facilitators retrieved from our included publications. The effectiveness measures included the proportions of those discharged from a malnutrition treatment programme who recovered, defaulted or died.

## Results

### Characteristics of the included literature

**Figure 1:**
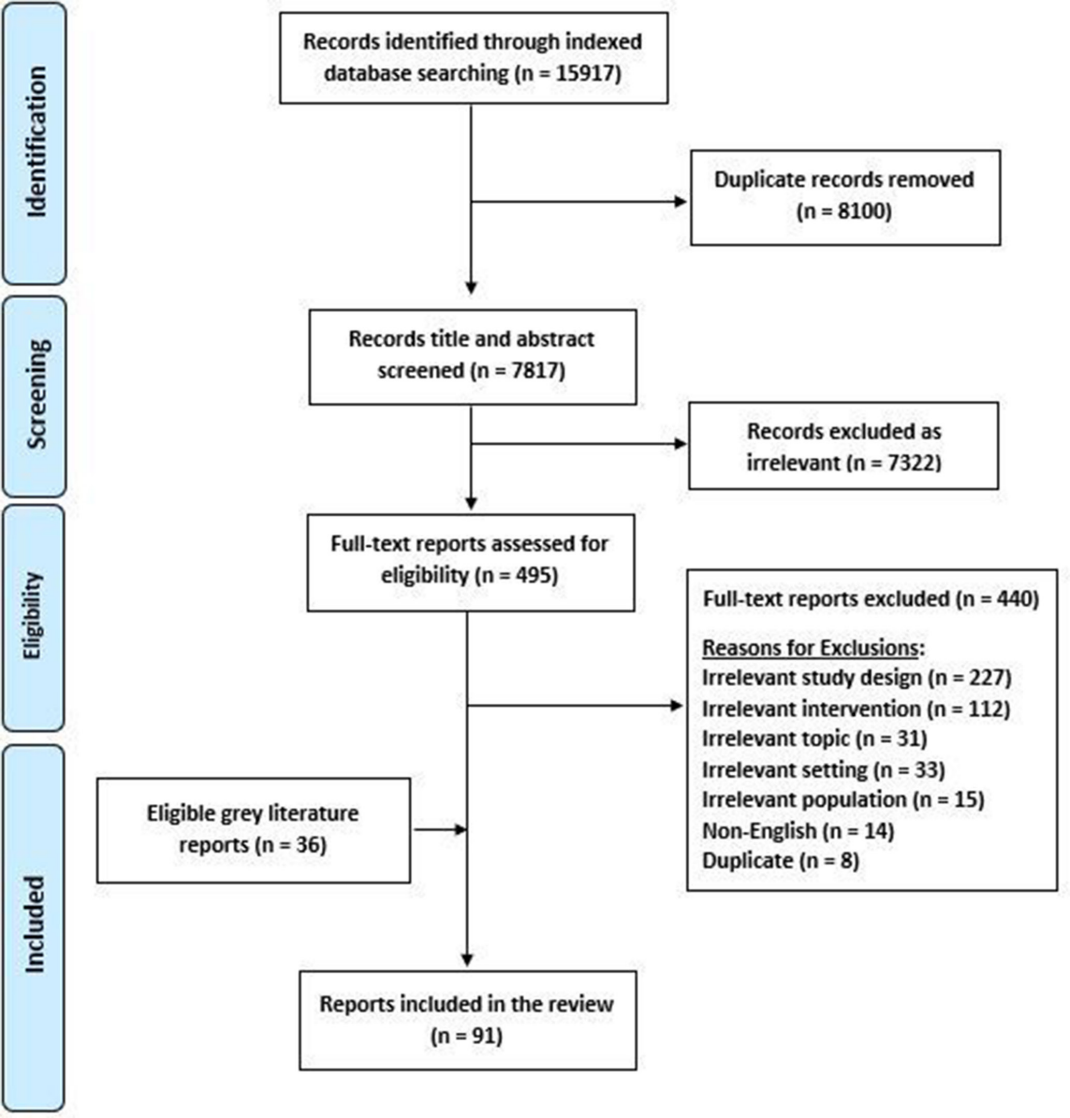
Preferred Reporting Items for Systematic Reviews and Meta-Analyses flow diagram.

We retrieved a total of 7817 unique citations from our indexed database search, and ultimately assessed 55^23–77^ of these as eligible for the review (figure 1). An additional 36^78–113^ eligible publications were identified from the grey literature and from the reference lists of relevant systematic reviews, resulting in a total of 91 publications being included in this review (online supplemental appendix 3).

**Figure 2:**
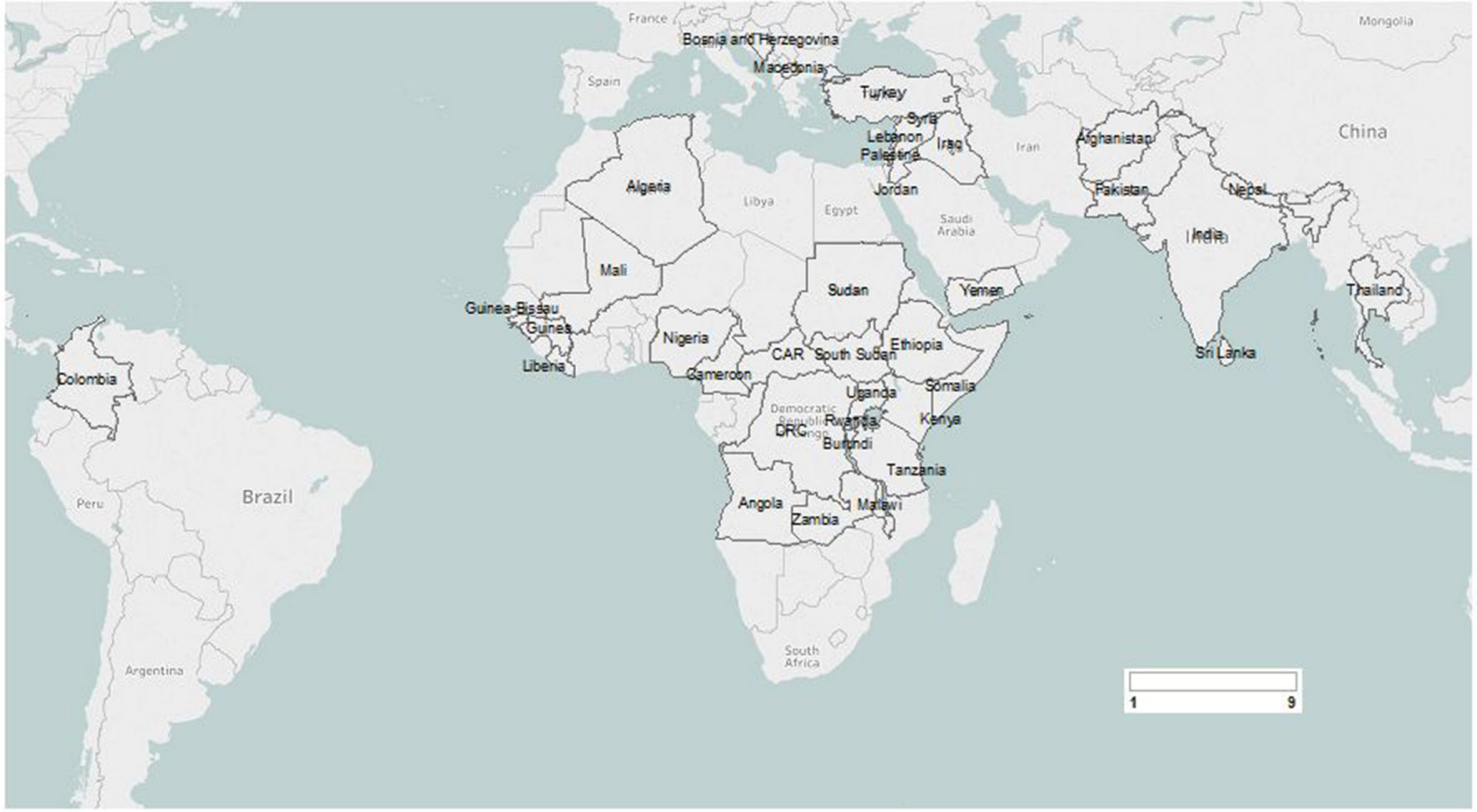
Geographic distribution of included publications.

Nearly half of the included publications (n=43, 47%) reported on nutrition interventions delivered in conflict-affected countries in sub-Saharan Africa and about one-third (n=31, 34%) in the Middle East and North African region (table 1). The country-level distribution of the included publications is illustrated in figure 2. Most publications (n=58, 64%) reported on interventions targeting children under 5 years of age, while 30% (n=27) reported on those targeting pregnant and lactating women. Refugee populations were the most targeted (n=53, 58%), while about one-third reported on internally displaced persons (IDPs) (n=33, 36%) and only about 16% (n=15) reported on intervention delivery among those not displaced. Intervention delivery was reported to occur in camp settings in two-thirds (n=63, 69%).

**Table 1:** Characteristics of included publications (n=91)

Geographic region*†	n
East Asia and Pacific	3
Europe and Central Asia	5
Latin America and the Caribbean	1
Middle East and North Africa	31
Sub-Saharan Africa	43
South Asia	9
*Publication type*	**n**
Non-research report	53
Observational study	32
Quasi-experimental study	2
Randomised controlled trial	4
*Target population type^†^*	**n**
All/general population	29
All women	4
Women of reproductive age	4
Pregnant and lactating women	27
Adolescents	10
Children under 5 years of age	58
*Displacement status of beneficiary population^†^*	**n**
Refugees	53
IDPs	33
Non displaced	15
Returning refugees	2
Host	10
Unreported	5
*Setting of displaced population^†^*	**n**
Camp	63
Dispersed	45
Unreported	2

*World Bank regions.

†Individual publications may contribute to multiple categories.

### Nutrition intervention delivery

**Figure 3:**
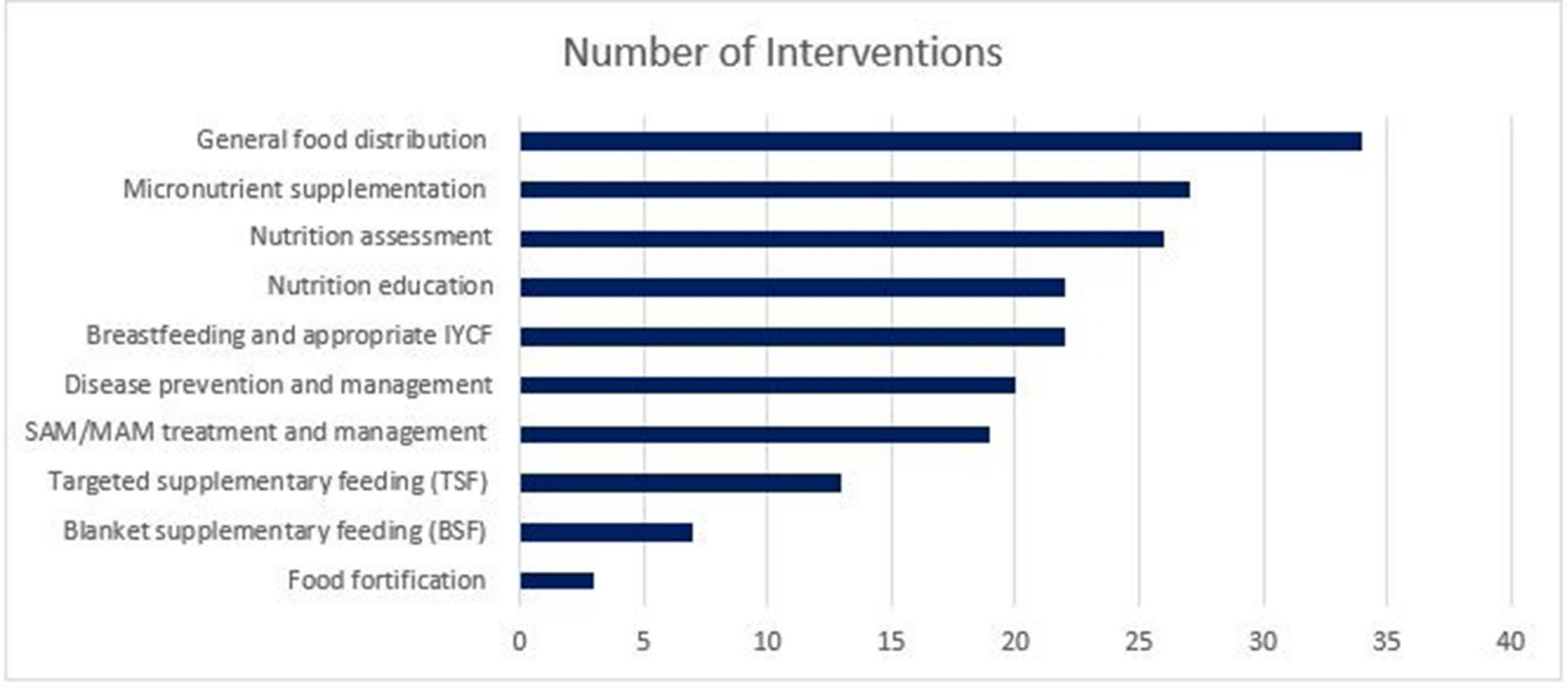
Reported nutrition interventions delivered to conflict-affected women and children. IYCF, infant and young child feeding.

Figure 3 presents the relative frequency of the various nutrition interventions captured in the included literature. Nutrition-specific interventions were implemented either alone, or in combination with other nutrition-specific or nutrition-sensitive interventions or activities. General food distribution (GFD) was the most frequently reported intervention, followed by micronutrient supplementation, nutrition assessment, nutrition education, breast feeding and appropriate feeding, disease prevention and management, supplementary feeding, severe acute malnutrition/moderate acute malnutrition (SAM/MAM) treatment, and food fortification.

We classified the individual interventions reported in the literature into broader categories, and we synthesise their components and delivery characteristics below.

#### Nutrition assessment

Twenty-six publications reported on mass screening for malnutrition as a component of other nutrition interventions.^23 25 27 29 32 49 51 53 69 73 77 79 83 84 87 88 92 96 100–102 104 107–109 111^ Of these, four were published between years 1990 and 2000,^23 29 32 77^ one between 2001 and 2010^51^ and 21 between 2011 and 2018.^25 27 49 53 69 73 79 83 84 87 88 92 96 100–102 104 107–109 111^ Ten studies conducted assessment in either hospitals or clinic or both,^27 29 32 83 84 101 102 104 108 109^ nine studies conducted assessments in mobile clinics which were established by government, non-government organisations (NGO), and United Nations (UN) agencies,^25 47 49 79 87 88 96 100 111^ four studies conducted assessment at home^23 51 53 107^ and three studies failed to report on it.^33 69 77^ Majority of the assessments were conducted at by NGO/UN staff.

#### Breastfeeding and appropriate infant and young child feeding

We found 22 publications reporting on the delivery of interventions to promote or support breast feeding or other infant and young child feeding (IYCF) interventions.^25 28 40 43 47 49 64 65 67 68 78 80 83 86 88 92 98 101 104 105 107 110^ Of these, five were published between years 2001 and 2010,^28 40 64 65 67^ and 17 between 2011 and 2018.^25 43 47 49 68 78 80 83 86 88 92 98 101 104 105 107 110^ Most commonly, breast feeding was promoted using an outreach approach of home visits conducted by health workers within camp settings.^49 64 65 78 98 105 107^ In reports on nutrition intervention delivery in Pakistan,^25^ Jordan^92^ and Somalia,^88^ mobile clinics were established by government and UN agencies in collaboration to promote breast feeding and sensitise refugee/internally displaced mothers on safe IYCF practices in and outside camp settings. Four publications reported on IYCF-related educational activities conducted among expectant/new mothers within hospital or clinic settings by community health workers (CHWs) or NGO/UN agency staff.^40 68 83 104^ In Macedonia, complementary foods were distributed through establishment of a food pipeline—a means to receive, store, and transport all donated food by multiple stakeholders.^28^ Among refugees and host populations in Jordan, the use of IYCF ‘caravans’^80^ and ‘safe havens’^110^ set up by NGOs to provide safe spaces for mothers to breastfeed infants was reported, as well as the distribution of breastfeeding shawls to lactating mothers to provide privacy.^80^

#### Disease prevention and management

Twenty publications reported on the delivery of interventions to either prevent and/or manage communicable disease among children under 5 years of age.^25 26 29 32 34 40 44 46 49 52 53 62 64–67 73 76 107 108^ Of these, six were published between years 1990 and 2000,^29 32 34 44 62 76^ nine between 2001 and 2010^25 26 40 46 52 64–67^ and five between 2011 and 2018.^49 53 73 107 108^ Some were conducted along with supplementary feeding as a part of nutrition rehabilitation programmes among refugee or internally displaced children under 5 years of age residing in camps or dispersed among local host populations in conflict settings.^25 26 29 32 34 40 44 46 49 53 64–66 76^ Seven publications reported on measles or cholera vaccination for children under-five in sub-Saharan African countries,^29 32 34 53 66 73 76^ delivered mostly at clinics/feeding centres by government or NGO/UN health workers. In India, vaccination was conducted by NGO/UN health workers as part of a measles outbreak response, using an outreach approach.^52^ Only one publication reported treatment of pregnant refugee women in Rwanda for malaria during antenatal care at UN/NGO clinic.^44^ Only one publication reported on the delivery of oral rehydration solution or zinc by CHWs to malnourished children under-five with diarrhoea, in a field-based trial among Afghan refugees residing along the border camps.^62^ Another publication reported on therapeutic treatment of diarrhoea in a hospital setting in Uganda among children under 2 years old.^108^ Deworming was delivered to children from 12 to 59 months of age as a part of facility/community-based nutrition rehabilitation programmes implemented in camps for refugees and IDPs by government health workers in Nepal, Sri Lanka and Yemen.^26 49 107^

#### Food fortification

Food fortification in conflict settings was not very commonly reported in the included literature. Only three publications reported on food fortification of which two were published between 2001 and 2010,^70 75^ and one between 2011 and 2018.^36^ Two reported on locally produced and milled wheat or maize flour fortification, distributed by NGO/UN staff within the food baskets for camp-based refugees in Afghanistan and Democratic Republic of the Congo (DRC).^70 75^ One recently published research study in Palestine reported the implementation of a fortification programme in the West Bank and Gaza, but how such fortified foods were delivered to women, children or households was not described.^36^

#### Micronutrient supplementation

Twenty-seven publications reported on the delivery of micronutrient supplementation interventions to women or children in conflict settings.^26 30 31 34 36 37 40 44 49 50 52 55 56 58 59 63–65 71 72 74 79 83 100 101 107 108^ Of which five were published between years 1990 and 2000,^31 34 44 58 74^ 10 between 2001 and 2010^37 40 52 55 56 59 64 65 72 79^ and 12 between years 2011 and 2018.^26 30 36 49 50 63 71 83 100 101 107 108^ Micronutrient supplementation in the form of powders was observed from year 2011 and onwards.^26 63 107 108^ We found six publications reporting iron and folic acid (IFA) supplementation being provided to pregnant or lactating women, among camp-based refugees or IDPs in Thailand,^30^ Rwanda,^44^ Palestine,^56^ Jordan,^79^ Lebanon^100^ and Yemen.^107^ IFA supplementation was delivered as part of routine antenatal care by health workers in the community-based primary health clinic/mobile clinics run by NGOs and/or local government. Two publications reported on thiamine supplementation for pregnant women living in camps along the Thai-Burma border^30^ and visiting antenatal clinics of Shoklo Malaria Research Unit.^59^ In Tanzania, camp-based refugees were provided with stainless steel cooking pots at community-based distribution points in a study to evaluate their effectiveness in reducing iron deficiency anaemia.^72^

Vitamin A supplementation for non-displaced/IDP/refugee children under-five living in West Bank and Gaza,^36 56^ Guinea Bissau,^59 65^ Nepal,^26^ India^52^ and Thailand^71^ was reported in seven publications, sometimes delivered as a part of measles treatment protocol implemented at outreach level by health workers.^52 64 65 71^ The delivery of multiple micronutrient (MMN) supplements for non-displaced/IDP/refugee women and children residing in Guinea Bissau,^59 64^ Afghanistan,^37^ Malawi,^58^ Nepal,^26^ Lebanon,^100^ Uganda^108^ and Yemen^107^ was reported in eight publications, distributed most commonly in powder, ready-to-use spread or cereal form through primary health centres or clinics. One publication reported on a randomised controlled trial in which pregnant women were provided with MMN tablets in Dadaab refugee camp in Kenya.^50^ Two publications reported on vitamin C supplementation interventions for refugee children in Ethiopia,^31^ and non-displaced children and adults in Afghanistan,^37^ although delivery modes were unreported. One publication reported on mass vitamin B complex supplementation provided to the general population by UN agencies in collaboration with local government as an emergency response to a pellagra outbreak in Malawi; where delivery site was unreported.^58^ Two publications reported on the delivery of micronutrient supplementation while providing nutrition rehabilitation through primary healthcare facilities to non-displaced children in Syria^101^ and refugee children in Lebanon.^83^

#### General food distribution

Thirty-four publications reported on GFD in conflict settings either through take home rations or hot cooked meal provision, or through cash or vouchers for food.^23 27–30 32 33 41–44 46 54 55 57–59 61 63 69 71 75 77 81 82 90 91 93–95 97 106 112 113^ Of these, eight were published between years 1990 and 2000,^23 29 32 33 44 54 58 77^ five between 2001 and 2010^28 46 55 59 75^ and 21 between 2011 and 2018.^27 30 41–43 57 61 63 69 71 81 82 90 91 93–95 97 106 112 113^ GFD in form of food vouchers/cash assistance was given from the year 2011 onwards.^27 41 43 82 91 95 106 112 113^ Four publications reported on specific food rations for pregnant women to meet extra nutrient requirements,^30 44 59 71^ delivered by staff from UN agencies. Six publications reported on food distribution interventions targeted at children under-five years of age,^23 27 31 32 46 64^ and one targeted at women of reproductive age,^63^ with food rations distributed at health centres, supplementary feeding centres or distribution points in the market. One publication reported on food rations delivered to everyone over the age of 15 years by NGO/UN staff in Uganda,^69^ but the delivery mode was unreported.

In publications describing those GFD interventions where food rations were provided to households, the delivery personnel involved were either NGO staff^28 41 61^ or were either not reported.^32^ In rural areas of northern Lebanon, community volunteers delivered hot meals multiple times per week to Syrian refugees through community kitchens.^81^ Two studies reported on delivering food rations to survivors of sexual and gender based violence, one study also provided ‘safe shelter boundaries’ during conflict to survivors of sexual and gender based violence.^78 94^ Only one record was found where military personnel distributed food rations, in Iraq, but delivery mode was unreported.^33^

The provision of food through cash or voucher distribution was reported in 10 publications.^27 42 43 82 91 95 97 106 112 113^ These included IDPs or refugees living in camps/dispersed settings in Lebanon, Turkey, Jordan, Syria, Central African Republic, Kenya and South Sudan and delivered by NGO/UN through print or electronic media (via mobile app/market-based ATMs) at homes/market/NGO clinics.

#### SAM/MAM treatment and management

A total of 19 publications reported on interventions to treat acute malnutrition on an inpatient basis.^24 25 40 49 60 66 73 79 80 83 85 89 98 100–102 104 109 111^ Of these publications, only two were published between 2001 and 2010,^40 66^ and 17 between 2011 and 2018.^24 25 49 60 73 79 80 83 85 89 98 100–102 104 109 111^ All nutrition interventions were conducted by local Ministry of Health (MOH) in collaboration with UN agencies/NGOs. The majority of the SAM treatment interventions included a combination of inpatient and community-based care of under-five children depending on the severity of malnutrition.^25 66 73 79 83 85 102 104 111^ SAM patients with complications were given inpatient care either in hospitals or therapeutic feeding centres, followed by provision of ready to use therapeutic food (RUTF), BP100 Plumpy nuts or F100 milk retrieved periodically from outpatient clinics in some cases, and onsite feeding at supplementary/outpatient therapeutic feeding centres in other cases.^25 49 60 66 80 83^ One study reported on mixing of RUTF with nutrient dense product (NRG-5) with milk or juice to mask up the foul taste of RUTF.^83^ The four main elements of the community-based management of acute malnutrition (CMAM) programme were management of MAM, management of SAM, inpatient management for SAM with medical complications and community outreach.^79 85 104^ The studies on CMAM programme were mostly published after the year 2015. In some conflict settings where pre-existing healthcare system was absent or unable to respond, temporary services and structures were established by UN agency/NGOs such as inpatient stabilisation centres for IDPs in South Sudan.^102^

#### Supplementary feeding

Twenty publications reported on supplementary feeding of which three were published between years 1990 and 2000,^31 74 76^ four between 2001 and 2010^37 56 64 65^ and 13 between 2011 and 2018.^26 49 73 79 80 83 89 93 99 101 102 104 109^ Seven included publications reported on the delivery of blanket supplementary feeding (BSF) interventions in conflict settings to prevent acute malnutrition.^31 37 49 74 93 99 101^ BSF was provided in addition to general food rations in three publications,^37 49 93^ while one publication reported on distribution of healthy baked snacks as thyme rolls and almond muffins,^99^ and one more publication reported on blanket distribution of ready to use supplementary food (RUSF) to internally displaced under five children as well as postnatal mothers in Syria through community-based clinics.^101^ Nearly all BSF programmes targeted children aged 6–59 months, predominantly in camp settings, but two publications reported BSF targeted at school-aged refugee children residing outside of camps and from the host community, in Somalia^93^ and Lebanon.^99^

Thirteen included publications reported on targeted supplementary feeding (TSF) interventions, aimed at treating MAM and preventing SAM.^26 56 64 65 73 76 79 80 83 89 102 104 109^ TSF was provided as a part of programme^102 104^ or as a standalone provision of RUSF,^83 89^ fortified local food^80^ or Super Cereal Plus.^80 109^ Most TSF interventions also focused on children aged 6–59 months in camp settings.

The majority of both blanket and TSF interventions were delivered either in supplementary feeding centre (SFC), in health centres or at distribution points in markets,^26 31 37 49 56 64 65 76 79 80 83 102^ mostly by CHWs and/or formal health workers,^26 37 49 56 64 65 73 76 83 104^ or by NGO/UN staff.^74 80 93 99 101 109^

#### Nutrition education

Twenty two publications reported on nutrition-focused education delivered alone or as a component of other nutrition interventions in conflict settings.^34 37 38 40 43 56 62 67 68 80 83 86 89 92 98 99 101 104 105 107 110 111^ Of these, two studies were published between years 1990 and 2000,^34 62^ four between 2001 and 2010,^37 40 56 67^ and 16 between 2011 and 2018.^38 43 68 80 83 86 89 92 98 99 101 104 105 107 110 111^ The majority of education initiatives focused on infant formula use, IYCF practices, water, sanitation and hygiene (WASH) promotion or on continued feeding of children during illness.^37 40 43 56 62 67 68 80 83 86 89 92 98 99 101 104 105 107 110 111^ One publication reported on educating women about infectious disease management among under-five children,^34^ and another on an emergency response during konzo outbreak where food safety-related education was provided.^38^ All nutrition education interventions were conducted in hospitals, health centres or NGO clinics by NGO staff, health workers or CHWs.

### Nutrition intervention coverage and effectiveness

Only eight publications^23 26 49 64 73 76 89 100^ reported on the coverage^23 26 49 64 76^ (table 2) or the effectiveness^49 64 73 76 89 100^ (table 3) of delivered nutrition interventions. Further 11 publications failed to report data on coverage and provided data in numbers only.^34 46 83 86 88 90–92 102 104 106^ Two publications reported on the outcomes of treatment programmes for SAM, reporting only on performance indicators from Yemen, and Lebanon based therapeutic feeding programmes.^89 100^ When we compared the results with Sphere recommendations, we found SFP conducted from March to September 1994 in Burundi based SFCs reported lower coverage (29.6%) than the recommended (>50%).^76^ The SAM treatment performance indicators also reported lower recovery rates (66.8%) than the recommended (>75%).^76^ Defaulters were also noted higher in proportion (29.2%) than the recommended (<15%) in Burundi.^76^ The same SFP reported coverage in Liberia, and DRC more than the minimum standard.^76^ The Bandim health project and humanitarian assistance in Guinea Bissau during 1998 and 1999 treated SAM children through community, and outreach approach.^64^ They also achieved almost minimum coverage (57%), and recovery rate (59.9%), while the defaulters were reported higher than the minimum (32%).^64^ Another reported coverage of similar programme in 1998 among refugees and non-displaced residents of Guinea Bissau^23^ and showed 87% coverage among refugees and 91% among residents.^23^ The same study reported on food distribution, which reported higher coverage among refugees (41%) as compared with non-displaced residents (16%).^23^ A camp based BSF and MNS programme implemented during 2008 and 2010 in Nepal reported above minimum coverage ranged between 95% and 98%.^26^ A large scale community-based nutritional status assessment at mobile clinics during the Nutrition Rehabilitation Programme in Sri Lanka also achieved high coverage (97.3% in camps; 86% in urban and rural areas) than the Sphere recommendations.^49^ This programme also reported higher recovery rate for SAM treatment using TSF alone (90%) and in combination of RUTF (94%) as compared with the treatment with only RUTF (42.5%).^49^ All these three treatments when used to treat MAM children, recovery rates were noted lower ranged between 32% and 50%.^49^ When all three SAM treatments were compared, the proportion of defaulters were noted lowest (0.9%) in the combined treatment with TSF and RUTF.^49^ The default rate for MAM treatment was unreported.^49^ Another large scale camp based feeding programme at SFCs in Tanzania and Kenya treated SAM children with recovery rates of 75%, and 78%, respectively.^73^ While the MAM recovery rates were reported as 76% in Tanzania, and 92% in Kenya.^73^ Within Yemen based TFP, the increase in recovery rates and transfers to OTP was likely due to the improved quality of care brought about by the training programme.^89^

**Table 2:** Reported coverage of nutrition interventions targeted to children under-five in conflict settings

Programme type; country (programme duration)	Intervention component for which coverage was measured	Delivery sites	Delivery personnel	Setting	Year coverage measured	Target population (N)	% (n) of target population covered
District Nutrition Rehabilitation Programme; Sri Lanka (2007–2009)^49^	Nutritional status assessment of children aged 6–59 months	Health clinics, weighing posts	Health workers	Camp	2007	3638	97.3 (3538)
				Camp, non-camp	2007	38 953	85.9 (33 461)
				Camp, non-camp	2008	43 221	85.8 (37 090)
Refugee Nutrition Programme; Nepal (2008–2010)^26^	Micronutrient powder for home fortification (Vita-Mix-It)	Health centre/clinic	Health and nutrition workers	Camp	2010	569	97.2 (557)
	Vitamin A supplements			Camp	2010	569	97.7 (556)
	Deworming			Camp	2010	569	95.1 (541)
Supplementary Feeding Programme; Liberia (1993–1994)^76^	Targeted supplementary feeding	Facility-based supplementary feeding centres	Community health workers	Non-camp	1994	–	69.9
Supplementary Feeding Programme; Burundi (March–September 1994)^76^	Targeted supplementary feeding	Facility-based and community-based supplementary feeding centres	Community health workers	Non-camp	1994	–	29.6
Supplementary Feeding Programme; DRC (1994–1995)^76^	Targeted supplementary feeding	Community-based supplementary feeding centres	Community health workers	Camp	1994	–	93.7
The Bandim Health Project; Guinea-Bissau (1998–1999)^64^	Targeted supplementary feeding, micronutrient supplementation	Health centres, households	Health workers	Rural	1999	433	57 (247)
International Committee for the Red Cross Food Distribution; Guinea-Bissau (June 1998)^23^	Targeted supplementary feeding to refugees	Households	NGO staff	Non-camp	1998	299	41 (123)
	Targeted supplementary feeding to residents (not displaced)					99	16 (16)
The Bandim Health Project; Guinea-Bissau (July and August 1998)^23^	Targeted supplementary feeding to refugees	Households	NGO staff	Non-camp	1998	98	87 (85)
	Targeted supplementary feeding to residents (not displaced)					267	91 (243)

DRC, Democratic Republic of the Congo; NGO, non-governmental organisation.

**Table 3:** Reported effectiveness of treatment interventions for acute malnutrition among children under-five in conflict settings

Outcome	Country	Setting	Intervention components	Delivery personnel	Delivery site	Children treated N	Recovered n (%)	Defaulted n (%)	Died n (%)
SAM treatment	Sri Lanka^49^	Camp	Therapeutic feeding including F75, F100 and RUTF (BP-100) in hospital and RUTF (BP-100) as outpatient; deworming; vitamin A supplementation	Paediatricians, health workers	Hospital, health centre	230	216 (93.9)	2 (0.9)	0 (0)
	Sri Lanka^49^	Camp, non-camp	Targeted supplementary feeding (HEBs)			1065	958 (89.9)	51 (4.8)	0 (0)
	Sri Lanka^49^	Camp, non-camp	Blanket supplementary feeding (CSB) (no therapeutic or targeted supplementary feeding)			306	130 (42.5)	86 (28.1)	0 (0)
	Kenya^73^	Camps	Therapeutic feeding	CHWs	SFCs	3014	2351 (78)	151 (5)	181 (6)
	Tanzania^73^	Camps	Therapeutic feeding	CHWs	SFCs	403	303 (75)	41 (10)	17 (4)
	Yemen^89^	Camps, rural	Therapeutic feeding	Doctors, nurses, medical students	Hospital-based therapeutic feeding centre	1103	78 (7.1)	196 (18)	60 (5.4)
MAM treatment	Sri Lanka^49^	Camp	Targeted supplementary feeding (HEBs), blanket supplementary feeding (CSB)	Health workers	Health centre	753	380 (50.5)	NR	0 (0)
	Sri Lanka^49^	Camp, non-camp	Targeted supplementary feeding (HEBs), blanket supplementary feeding (CSB)			6970	3110 (44.6)	NR	0 (0)
	Sri Lanka^49^	Camp, non-camp	Blanket supplementary feeding (no targeted supplementary feeding)			4857	1571 (32.3)	NR	0 (0)
	Kenya^73^	Camps	Targeted supplementary feeding	CHWs	SFCs	37 741	34 722 (92)	1737 (5)	0 (0)
	Tanzania^73^	Camps	Targeted supplementary feeding	CHWs	SFCs	2158	1641 (76)	195 (9)	0 (0)
GAM treatment	Guinea Bissau^64^	Camps	Targeted supplementary feeding	Health workers	Health centre	247	148 (59.9)	70 (32)	2 (0.9)
	Lebanon^100^	Camps, rural	Therapeutic feeding	Doctors, nurses, CHWS	Hospital, health centre	519	412 (79.2)	24 (4.6)	NR
	Liberia^76^	Rural	Targeted supplementary feeding	CHWs	SFCs	12 259	9967 (81.3)	1923 (15.7)	50 (0.4)
	Burundi^76^	Rural	Targeted supplementary feeding	CHWs	SFCs	9197	6144 (66.8)	2682 (29.2)	63 (0.7)
	DRC^76^	Camps	Targeted supplementary feeding	CHWs	SFCs	18 767	14 826 (79)	2139 (11.4)	33 (0.2)

CHWs, community health workers; CSB, corn–soya blend; GAM, global acute malnutrition; HEBs, high-energy biscuits; MAM, moderate acute malnutrition; RUTF, ready to use therapeutic food; SAM, severe acute malnutrition; SFCs, supplementary feeding centres.

### Barriers to and facilitators of nutrition intervention delivery

Specific delivery barriers that we identified from the literature are presented in table 4. Insufficient resources and ongoing insecurity were key barriers recurring in the literature. Multiple publications reported on supply shortages of important commodities, especially of RUTF and micronutrient supplements, as well as insufficient human resources and limited funding. Humanitarian actors faced multiple security and accessibility issues due to ongoing conflict, collapsed healthcare systems, and damaged infrastructure, while limited inter-cluster coordination and lack of coordination between humanitarian partners posed additional delivery challenges. Population movements in and out of camps made it difficult to reach vulnerable populations and provide follow-up care. Gender bias and negative sociocultural practices (genital mutilation, early marriages, child labour) also hindered in intervention delivery. Outcome assessment by unskilled staff, security concerns, reporting errors, small sample size, and less rigorous methods resulted in poor reporting of coverage and effectiveness outcomes in the included studies.

**Table 4:** Nutrition intervention barriers and facilitators

	Themes	Specific examples	Countries	Interventions
Barriers	*Insufficient coordination*	Limited inter-cluster coordination^88 102 106 108^ Lack of cross border cooperation^67^	Kenya,^106^ Somalia,^88^ South Sudan,^102^ Tanzania,^67^ Uganda^108^	Nutrition assessment,^67 88 102 108^ breast feeding and appropriate IYCF,^67 88^ disease prevention and management,^67 108^ micronutrient supplementation,^108^ general food distribution,^106^ SAM/MAM treatment,^102^ supplementary feeding^102^
	*Insufficient resources*	Inadequate/irregular supplies of commodities^39 41 48 49 77 82 83 98 101 112^ Logistical constraints^29 79 99 100 112^ Limited human resources^83 89^ Limited funding^42 51 54 60 78 91 95^	Burundi,^48^ Somalia,^39^ Jordan,^41 51 79 95^ Syria,^41 42 101^ Sri Lanka,^49^ Bosnia and Herzegovina,^54 77^ Lebanon,^82 83 91 99 100^ Yemen,^89^ South Sudan,^98 112^ Democratic Republic of the Congo (DRC),^29^ Uganda,^60^ Palestine^78^	Nutrition assessment,^29 49 51 77 79 83 100 101^ breastfeeding and appropriate IYCF,^48 49 78 98^ disease prevention and management,^29 39 49 100^ micronutrient supplementation,^49 83^ general food distribution,^29 41 42 54 77 82 91 95 112^ SAM/MAM treatment,^49 60 79 83 89 100 101^ supplementary feeding,^49 79 83 89 99 101^ nutrition education^83 89 98 99 101^
	*Ongoing conflict situation*	Security and access concerns^29 41 60 80 84 89–91 94 101 102^	Lebanon,^91^ South Sudan,^102^ Yemen,^89^ Afghanistan,^84^ Syria,^101^ Jordan,^41 80^ DRC,^29^ Uganda,^60^ Colombia^94^	Nutrition assessment,^29 84 101 102^ breastfeeding and appropriate IYCF,^80^ disease prevention and management,^29^ micronutrient supplementation,^101^ general food distribution,^29 41 91 94^ SAM/MAM treatment,^60 80 89 101 102^ supplementary feeding,^89 101 102^ nutrition education^80 89 101^
	*Limited population adherence*	Limited cooperation from beneficiaries^63 80 86 92 93 107 110 111^ Population movement^76 102^ Gender bias or negative socio-cultural practices^93^	South Sudan,^102^ Jordan,^80 92^ Lebanon,^86 110^ DRC,^76 102^ Somalia,^93^ Burundi,^76^ Liberia,^76^ Kenya,^63^ Yemen^107^	Nutrition assessment,^92 102 107 111^ breastfeeding and appropriate IYCF,^80 86 92 110^ disease prevention and management,^76 107^ micronutrient supplementation,^63 107^ general food distribution,^63 93^ SAM/MAM treatment,^80 102 111^ supplementary feeding,^80 93 102^ nutrition education^80 86 92 107 110 111^
	*Poor outcome reporting*	Unskilled staff^38^ Reporting errors^62 73^ Small sample size/Sampling bias^42 53^ Security issues^42^	Cameroon,^38^ Kenya,^73^ Pakistan,^62^ Nigeria,^53^ Syria,^42^ Tanzania^73^	Nutrition assessment,^53 73^ disease prevention and management^62 73^ general food distribution,^42^ SAM/MAM treatment,^73^ supplementary feeding,^73^ nutrition education^38 62^
Facilitators	*Effective monitoring system*	Established nutrition surveillance system^64 65 83–85 101 109 112^	*Lebanon,*^83^ *Sudan,*^85^ Kenya,^64^ Guinea-Bissau,^64 65^ Afghanistan,^84^ Syria,^101^ Jordan,^109^ South Sudan^112^	Nutrition assessment,^83 84 101 109^ breastfeeding and appropriate IYCF,^64 65^ *micronutrient supplementation*,^64 65 83 101^ *general food distribution,*^64 112^ SAM/MAM treatment,^83 85 101 109^ supplementary feeding,^65 83 101 109^ nutrition education^83 101^
	*Multi-sector programming*	Inter-Cluster Coordination Group (ICCG Somalia)^88^ Nutrition cluster in partnership with the health and WASH clusters^25 88^ Integration of services through public primary healthcare (PHC) centres^31 39 40 48 49 66^ Nutrition services integration into public education system^99^	Burundi,^48^ Somalia,^39 88^ Sri Lanka,^49^ Pakistan,^25^ Ethiopia,^31^ Guinea-Bissau,^40^ DRC,^66^ Lebanon^99^	Nutrition assessment,^25 49 88^ breastfeeding and appropriate IYCF,^25 48 49 66 88^ disease prevention and management,^25 39 40 49^ micronutrient supplementation,^31 40 49 66^ SAM/MAM treatment,^25 40 49^ supplementary feeding,^31 49 66 99^ nutrition education^40 99^
	*Adoption of guidelines/evidence-based approaches*	National plan for scaling up CMAM^79 85 104^ ‘Scaling Up Nutrition’ movement^43^	Jordan,^79^ Sudan,^85^ Ethiopia,^85 104^ Central African Republic^43^	Nutrition assessment,^79 104^ micronutrient supplementation,^79^ general food distribution,^43^ SAM/MAM treatment,^79 85 104^ supplementary feeding,^79 104^ nutrition education^43 104^
	*Advocacy and social mobilisation*	Established village development committees^108^ Community networking^47 92^ Community mobilisation^38 70 75 79 86 87 100 105^ Communication for Development (C4D) approach^103^	Uganda,^108^ Jordan,^47 79 92^ Cameroon,^38^ Zambia,^70 75^ Angola,^75^ Afghanistan,^75^ Lebanon,^86 87 100^ South Sudan,^105^ Mali^103^	Nutrition assessment,^79 87 92 100 108^ breastfeeding and appropriate IYCF,^47 86 92 105^ disease prevention and management,^100 108^ food fortification,^70 75^ micronutrient supplementation,^79 100 108^ general food distribution,^75^ SAM/MAM treatment,^79 100^ supplementary feeding,^79^ nutrition education^38 86 92 105^
	*Capacity building of workforce*	Task shifting/sharing^85^ Training of trainers^68 85 89^	Sudan,^85^ Yemen,^89^ South Sudan^68^	SAM/MAM treatment,^85 89^ supplementary feeding,^89^ nutrition education^68 89^
	*Innovative technology use*	Biometric technology for mobile phone cash transfer (M-PESA)^91 95^	Lebanon,^91^ Jordan^95^	Food vouchers/cash provision^91 95^

CMAM, community-based management of acute malnutrition; IYCF, infant and young child feeding; SAM/MAM, severe acute malnutrition/moderate acute malnutrition; WASH, water, sanitation and hygiene.

Effective social mobilisation, monitoring and surveillance, and integration of nutrition services into other sectors were key delivery facilitators (table 4). Local community members, religious leaders and established community networks were leveraged to effectively deliver nutrition interventions. The establishment of nutrition surveillance systems was found to be effective in strengthening local monitoring systems. A multi-sectoral programming approach was reported in multiple instances, with the nutrition cluster collaborating with the health and WASH clusters to facilitate delivery of comprehensive nutrition-specific and sensitive interventions, and interventions being integrated into local healthcare systems. Some national-level programmes adopted evidence-based guidelines to scale up nutrition interventions through community-based approaches, especially for the acute malnutrition. There were several examples of intensive training and capacity building of workers, as well as task shifting, implemented to improve coverage of acute malnutrition and IYCF interventions.

## Discussion

**Figure 4:**
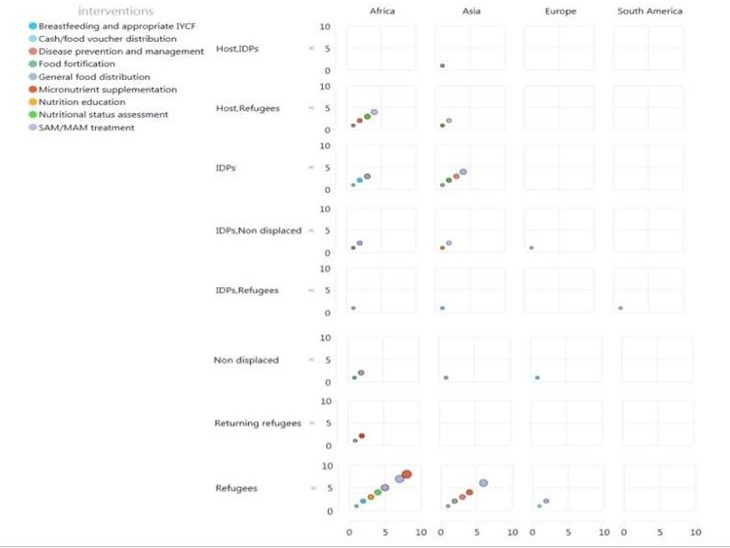
Summary of evidence. IDP, internally displaced person; IYCF, infant and young child feeding; SAM/MAM, severe acute malnutrition/moderate acute malnutrition.

We identified 91 publications from 1990 and 2018 that described the delivery of nutrition interventions to women and children affected by armed conflict in LMICs, mostly reported in African region. Less than half of the included publications reported on research findings, and nearly 40% were sourced from the grey literature. Studies published between year 1990 and 2000, majorly focused on GFD and disease prevention and management. Studies published between year 2001 and 2010 also focused on disease prevention and management and on micronutrient supplementation, and studies published after 2011 focused on nutrition assessment, GFD and SAM/MAM treatment. GFD, micronutrient supplementation and nutrition assessment were the most frequently reported interventions, with most publications reporting on intervention delivery to refugee populations in camp settings and using community-based approaches (figure 4).^114^ Limited data on intervention coverage or effectiveness were captured from the included literature, preventing inferences to be drawn about how these vary by different delivery approaches in conflict settings. Very rarely were quantitative estimates reported, but delivery mechanisms and barriers and facilitators were more comprehensively described in the grey compared with the indexed literature. Insufficient resources, including nutritional commodity shortages, security concerns due to ongoing conflict, limited inter-cluster coordination, and difficulty accessing and following beneficiaries up due to population movements and sometimes limited cooperation were key delivery barriers. Community advocacy and social mobilisation, effective monitoring, and integration of nutrition and other sector interventions and services were key delivery facilitators.

Our results yield important insights about the nutrition delivery and important gaps. It is evident that much of the documentation of nutrition interventions and programmes implemented in conflict and likely in emergency settings more generally, exists in the grey literature generated by UN agencies, NGOs and other humanitarian implementers actively working in the field rather than from government reports and indexed literature. Second, the limited number of included studies and variation in population behaviour and in context such as country, underlying health system, disease outbreak, and type and severity of conflict has curtailed us from studying the impact of nutrition intervention on women in conflict setting in greater depth.

In addition, much of the literature focused on nutrition interventions delivered to camp-based refugees, with relatively little reported on populations displaced or non-displaced populations. It is difficult to know whether the lack of reporting on non-camp and non-displaced populations reflects actual nutrition intervention delivery patterns on the ground, or rather a failure to document.

With respect to the types of interventions, the relatively higher frequency of reporting on food distribution and the management of acute malnutrition is understandable, given the high prevalence of food insecurity and the high morbidity and mortality burden of malnutrition. It was somewhat surprising to find relatively little on the delivery of IYCF interventions, though there were some examples of innovative practices to provide safe spaces for women to breast feed. Moreover, we captured just as many publications reporting on infant formula distribution as on breastfeeding promotion interventions.

We also found that many publications reported on nutrition intervention delivery at household level or through outreach approaches. We also note that the CMAM has been adopted as national policy by several governments including Ethiopia, Jordan and Sudan. Given these experiences, the humanitarian nutrition sector may be particularly well-placed to further innovate and test community-based approaches that might overcome or circumvent the specific implementation challenges across sectors.

In context to barriers of delivery; destruction of health facilities, targeted attacks on facilities and health workers, as well as disruption of supply chains were key issues, which further added to the existing weak governance and healthcare system infrastructure. Thus, the actors should identify effective strategies for delivering interventions through planning which must addresses the security concerns of health service providers and beneficiaries. Moreover, gathering support and acceptance from local influencers and communities, including local authorities, appears to be critical, while maintaining the perception of their impartiality and neutrality.

Finally, literature reports on multi-sectoral programming approach with the nutrition cluster collaborating with the health and WASH clusters, but has failed to report its integration with early childhood development and mental health, which is an emerging issue. Moreover, there is no data on gender equality and social inclusion. Thus, future studies should untake gender analyses, to investigate the gendered differences in access, needs and uptake of healthcare services.

This systematic review of the literature is the first, to our knowledge, to focus on the delivery of nutrition interventions in armed conflict settings, and thus makes a novel and important contribution to the field. However, in addition to the limitations inherent in the existing literature itself, discussed above, we must also acknowledge that by restricting our review only to publications published in English, and by undertaking a comprehensive but not exhaustive search of the grey literature, we have inevitably excluded other relevant publications that may have provided different information from what we have captured presently.

## Conclusion

There is very little information on achieved coverage or effectiveness of nutrition interventions delivered in conflict settings; more (and more rigorous) evaluation of different delivery approaches is needed, including outside of camps and for preventive as well as curative nutrition interventions. The humanitarian nutrition sector may be particularly well-placed to advance the field with respect to community-based intervention delivery in conflict contexts, and has effective, existing networks to widely disseminate the important evidence that it could generate.

## Data Availability

Data are available upon request. Data extracted from publications retrieved from the indexed and grey literature are available from the corresponding author upon reasonable request.
