# Supplementary material for: Delivering nutrition interventions to women and children in conflict settings: a systematic review
Source: BMJ Glob Health. 2021 Apr 8;6(4):e004897. doi: 10.1136/bmjgh-2020-004897 (PMC8039262; doi:10.1136/bmjgh-2020-004897)
Supplement: Supplementary data [file bmjgh-2020-004897supp001.pdf]

Appendix 1. PRISMA checklist

Section/topic	#	Checklist item	Reported on page #
**TITLE**			
Title	1	Identify the report as a systematic review, meta-analysis, or both.	1
**ABSTRACT**			
Structured summary	2	Provide a structured summary including, as applicable: background; objectives; data sources; study eligibility criteria, participants, and interventions; study appraisal and synthesis methods; results; limitations; conclusions and implications of key findings; systematic review registration number.	3
**INTRODUCTION**			
Rationale	3	Describe the rationale for the review in the context of what is already known.	5
Objectives	4	Provide an explicit statement of questions being addressed with reference to participants, interventions, comparisons, outcomes, and study design (PICOS).	5
**METHODS**			
Protocol and registration	5	Indicate if a review protocol exists, if and where it can be accessed (e.g., Web address), and, if available, provide registration information including registration number.	5
Eligibility criteria	6	Specify study characteristics (e.g., PICOS, length of follow-up) and report characteristics (e.g., years considered, language, publication status) used as criteria for eligibility, giving rationale.	5-6
Information sources	7	Describe all information sources (e.g., databases with dates of coverage, contact with study authors to identify additional studies) in the search and date last searched.	5
Search	8	Present full electronic search strategy for at least one database, including any limits used, such that it could be repeated.	Appendix 2
Study selection	9	State the process for selecting studies (i.e., screening, eligibility, included in systematic review, and, if applicable, included in the meta-analysis).	5-6
Data collection process	10	Describe method of data extraction from reports (e.g., piloted forms, independently, in duplicate) and any processes for obtaining and confirming data from investigators.	6
Data items	11	List and define all variables for which data were sought (e.g., PICOS, funding sources) and any assumptions and simplifications made.	5-6

Risk of bias in individual studies	12	Describe methods used for assessing risk of bias of individual studies (including specification of whether this was done at the study or outcome level), and how this information is to be used in any data synthesis.	Not applicable
Summary measures	13	State the principal summary measures (e.g., risk ratio, difference in means).	6
Synthesis of results	14	Describe the methods of handling data and combining results of studies, if done, including measures of consistency (e.g., I^2) for each meta-analysis.	6
Risk of bias across studies	15	Specify any assessment of risk of bias that may affect the cumulative evidence (e.g., publication bias, selective reporting within studies).	Not applicable
Additional analyses	16	Describe methods of additional analyses (e.g., sensitivity or subgroup analyses, meta-regression), if done, indicating which were pre-specified.	Not applicable
**RESULTS**			
Study selection	17	Give numbers of studies screened, assessed for eligibility, and included in the review, with reasons for exclusions at each stage, ideally with a flow diagram.	6
Study characteristics	18	For each study, present characteristics for which data were extracted (e.g., study size, PICOS, follow-up period) and provide the citations.	6-7
Risk of bias within studies	19	Present data on risk of bias of each study and, if available, any outcome level assessment (see item 12).	Not applicable
Results of individual studies	20	For all outcomes considered (benefits or harms), present, for each study: (a) simple summary data for each intervention group (b) effect estimates and confidence intervals, ideally with a forest plot.	Not applicable
Synthesis of results	21	Present results of each meta-analysis done, including confidence intervals and measures of consistency.	7-18 (Meta-analysis not done)
Risk of bias across studies	22	Present results of any assessment of risk of bias across studies (see Item 15).	Not applicable
Additional analysis	23	Give results of additional analyses, if done (e.g., sensitivity or subgroup analyses, meta-regression [see Item 16]).	Not applicable
**DISCUSSION**			
Summary of evidence	24	Summarize the main findings including the strength of evidence for each main outcome; consider their relevance to key groups (e.g., healthcare providers, users, and policy makers).	19

Limitations	25	Discuss limitations at study and outcome level (e.g., risk of bias), and at review-level (e.g., incomplete retrieval of identified research, reporting bias).	19-20
Conclusions	26	Provide a general interpretation of the results in the context of other evidence, and implications for future research.	20
**FUNDING**			
Funding	27	Describe sources of funding for the systematic review and other support (e.g., supply of data); role of funders for the systematic review.	21

From: Moher D, Liberati A, Tetzlaff J, Altman DG, The PRISMA Group (2009). Preferred Reporting Items for Systematic Reviews and Meta-Analyses: The PRISMA Statement. PLoS Med 6(7): e1000097. doi:10.1371/journal.pmed1000097

For more information, visit: **www.prisma-statement.org**.

Appendix 2. MEDLINE search syntax

Conflict-related terms

1. disasters/ or emergencies/ or mass casualty incidents/
2. disaster victims/
3. ((disaster or disasters or catastrophe or catastrophes) adj5 (environ* or human or manmade or "man made" or nature or natural or weather)).tw,kf.
4. ("mass casualty" or "mass casualties" or "mass fatalities" or "mass fatality").tw,kf.
5. ((crisis or crises) adj5 (environ* or human or manmade or "man made" or nature or natural or weather)).tw,kf.
6. "warfare and armed conflicts"/ or armed conflicts/ or warfare/ or biological warfare/ or bioterrorism/ or chemical warfare/ or chemical terrorism/ or nuclear warfare/ or psychological warfare/ or war crimes/ or ethnic cleansing/ or genocide/ or holocaust/ or war exposure/ or war-related injuries/
7. afghan campaign 2001-/ or gulf war/ or iraq war, 2003-2011/
8. ("afghan campaign" or "armed conflict" or "armed conflicts" or "gulf war" or "iraq war" or "war time" or "wartime").tw,kf.
9. ((armed or zone or political or civil) adj3 (conflict or conflicts or attack or attacks or war or wars or "no fly")).tw,kf.
10. ("war related injuries" or "war related traumas" or "war related injury" or "war related trauma").tw,kf.
11. ("militant group" or "militant groups" or "militant organization" or "militant organizations" or "militant organisation" or "militant organisations").tw,kf.
12. ("biological terrorism" or bioterrorism or biowarfare or "chemical terrorism" or "ethnic cleansing" or "ethnic cleansings" or "gas poisoning" or genocide or holocaust or holocausts or "nuclear terrorism" or "war exposure" or "war exposures").tw,kf.
13. Disaster Medicine/
14. disease outbreaks/
15. Emergency Medical Services/
16. ((emergency or emergencies) adj5 (environ* or human or manmade or "man made" or nature or natural or weather)).tw,kf.
17. Starvation/
18. (famine or famines or starvation or starvations).tw,kf.
19. cyclonic storms/ or droughts/ or floods/ or tornadoes/ or tidal waves/
20. avalanches/ or earthquakes/ or landslides/ or tidal waves/ or tsunamis/ or volcanic eruptions/
21. (avalanche or avalanches or cyclone or cyclones or drought or droughts or earthquake or earthquakes or flood or flooded or flooding or floods or hurricane or hurricanes or landslide or landslides or "land slide" or "land slides" or mudslide or mudslides or "mud slide" or "mud slides" or storm or storms or tornado or tornadoes or tsunami or tsunamis or typhoon or typhoons or "volcanic ash" or "volcanic eruption" or "volcanic eruptions" or "volcanic gases").tw,kf.
22. refugees/
23. (evacuee or evacuees or refugee or refugees or squatter or squatters or transients).tw,kf.
24. relief work/ or rescue work/
25. ((rescue or relief or aid) adj (plan or plans or activity or activities or agency or agencies)).tw,kf.
26. ("aid plan" or "aid work" or "relief plan" or "relief work" or "rescue plan" or "rescue work").tw,kf.
27. ((staff or staffs or worker or workers) adj3 (relief or aid)).tw,kf.
28. (humanitarian assistance or humanitarian assistances or relief work or relief works).tw,kf.
29. (humanitarian adj2 (aid or response or relief or crisis or crises or emergency or emergencies or disaster or disasters)).tw,kf.
30. Altruism/

31. (humanitarianism or altruism).tw,kf.
32. ("displaced children" or "displaced families" or "displaced family" or "displaced individuals" or "displaced internally" or "displaced men" or "displaced people" or "displaced peoples" or "displaced person" or "displaced persons" or "displaced population" or "displaced populations" or "displaced women" or "forced displacement" or "forced displacements" or "internal displaced" or "internal displacement" or "internally displaced" or "population displaced" or "population displacement").tw,kf.
33. (((camp or camps) and displac*) or "protected village*").tw,kf.
34. (victim or victims).tw,kf.
35. rubble.tw,kf.
36. or/1-35

Population-related terms

37. adolescent/ or young adult/
38. (adolescence or adolescent or adolescents or teen* or youth or youths or "young adult" or "young adults").tw,kf.
39. Pregnant Women/
40. exp pregnancy/
41. (expectant or expectancy or gravid* or pregnant or pregnancies or pregnancy).tw,kf.
42. ("mother to be" or "mothers to be").tw,kf.
43. (prenatal or "pre natal").mp.
44. (perinatal or "peri natal").mp.
45. ((trimester or trimesters) adj3 (first or second or mid or third or final or "1st" or "2nd" or "3rd")).tw,kf.
46. (midtrimester or midtrimesters or "early placental phase" or "early placental phases").tw,kf.
47. exp Delivery, Obstetric/
48. ((labor or labour) adj5 (birth* or breech or childbirth or childbirths or complicat* or difficult or early or easy or induce* or induction or late or obstetric* or onset or pregnan* or present*)).tw,kf.
49. parturients.tw,kf.
50. (birth or births or childbirth or childbirths or parturition or parturitions).tw,kf.
51. ("abdominal deliveries" or "abdominal delivery" or "c-section" or "c-sections" or caesarean or caesareans or cesarean or cesareans or "postcesarean section" or "postcaesarean section").tw,kf.
52. exp Abortion, Induced/
53. (abortion or abortions or embryotomies or embryotomy or "postconception fertility control").tw,kf.
54. ((pregnancy or pregnancies) adj3 terminat*).tw,kf.
55. "sexually active".tw,kf.
56. child/ or child, preschool/ or infant/ or infant, newborn/ or infant, low birth weight/ or infant, small for gestational age/ or infant, very low birth weight/ or infant, extremely low birth weight/ or infant, postmature/ or infant, premature/ or infant, extremely premature/
57. (infan* or newborn* or "new born*" or neonat* or baby* or babies or toddler* or boy or boys or boyfriend or boyhood or girl* or kid or kids or child* or pediatric* or paediatric* or peadiatric* or prematur* or preterm*).mp. or school*.tw.
58. refugees/
59. (refugee or refugees).tw,kf.
60. or/37-59
61. 36 and 60

Nutrition-related terms

62. Arm/ and Anthropometry/
63. ((arm or arms or midarm or midarms) adj3 (anthropometr* or circumference)).tw,kf.

64. muac.tw,kf.
65. (Body Weight/ or Body Height/) and Anthropometry/
66. ("height to weight ratio" or "height weight ratio" or "height for age" or "weight for age" or "weight for height" or "weight for length" or "weight to height ratio" or "haz" or "waz" or "whm" or "whz" or stunting or stunted or underweight or "under weight").tw,kf.
67. Infant Nutritional Physiological Phenomena/ or Child Nutritional Physiological Phenomena/ or Adolescent Nutritional Physiological Phenomena/
68. ("adolescent nutrition physiology" or "adolescent nutritional physiological phenomena" or "adolescent nutritional physiological phenomenon" or "adolescent nutritional physiology" or "child nutrition physiology" or "child nutritional physiological phenomena" or "child nutritional physiology" or "child nutritional physiology phenomena" or "child nutritional physiology phenomenon" or "infant nutrition physiology" or "infant nutritional physiological phenomena" or "infant nutritional physiological phenomenon").tw,kf.
69. milk, human/ or infant formula/ or breast feeding/ or breast milk expression/
70. ("breast feeding" or "breast milk" or "breastmilk or breast pumping or breast pumpings or breastfed or breastfeeding or artificial milk or baby formula or baby formulas" or "formula feeding" or "formula milk" or "formulated milk" or "infant formula" or "infant formulas" or "milk formula" or similac or "synthetic milk").tw,kf.
71. Milk Banks/
72. ("milk bank" or "milk banks" or "milkbank" or "milkbanks").tw,kf.
73. ((transfer* or voucher or vouchers) adj15 (cash or money)).tw,kf.
74. (feeding centre or feeding centres or feeding center or feeding centers).tw,kf.
75. Food Assistance/
76. ("food aid" or "food assistance" or "food stamp" or "food stamps" or "snap program" or "snap programs" or "supplemental nutrition assistance program" or "supplemental nutrition assistance programme" or "wic program" or "wic programs").tw,kf.
77. Food, Fortified/
78. ("enriched food" or "enriched foods" or "food fortification" or "fortified food" or "fortified foods" or "supplemented food" or "supplemented foods").tw,kf.
79. nutrition therapy/ or diet therapy/
80. ("diet modification" or "diet modifications" or "diet therapies" or "diet therapy" or "diet treatment" or "diet treatments" or "dietary modification" or "dietary modifications" or "dietary therapy" or "dietary treatment" or "dietary treatments" or "nutrition therapy").tw,kf.
81. "food relief".tw,kf.
82. "fortified milk".tw,kf.
83. "general food distribution".tw,kf.
84. "general ration distribution".tw,kf.
85. ((energy or protein or cereal) adj (bar or bars or biscuit* or cookie* or milk or food or foods)).tw,kf.
86. Hunger/
87. (hunger or famine or hungry).tw,kf.
88. Iodine/df
89. "iodine deficienc*".tw,kf.
90. Goiter, Endemic/
91. (goitre or goitres or goiter or goiters).tw,kf.
92. Kwashiorkor/
93. ("amino acid starvation" or "aminoacid starvation" or kwashiokor or kwashiorkor or kwashiorkors or kwasiorkor or "protein deprivation" or "protein malnutrition" or "protein starvation").tw,kf.
94. Lipids/ and Dietary Supplements/
95. ((("lipid* based" or energy) adj3 supplement*) or plumpy or "lns" or nutrispread or "nutri spread").tw,kf.
96. Malnutrition/ or Protein-Energy Malnutrition/ or Severe Acute Malnutrition/

97. ("deficient nutrition" or "malnourished" or "malnourishment" or "malnourishments" or "malnutrition" or "marasmus" or "nutritional deficiencies" or "nutritional deficiency" or "under fed" or "under feeding" or "under nourishment" or "under nutrition" or "underfed" or "underfeeding" or "undernourishment" or "undernutrition").tw,kf.
98. Micronutrients/df
99. ((micronutrient or micronutrients or "trace element" or "trace elements" or "trace mineral" or "trace minerals") adj5 (deficienc* or "defective diet" or "deficient diet" or "diet insufficienc*" or "dietary insufficienc*" or "nutritional deficit*")).tw,kf.
100. (Micronutrients/ or Trace Elements/) and (Food, Fortified/ or Dietary Supplements/)
101. ((micronutrient or micronutrients or "trace element" or "trace elements" or "trace mineral" or "trace minerals") adj10 (diet* or nutrition*) adj3 supplement*).tw,kf.
102. ("micronutrient powder" or "micronutrient powders" or mnp).tw,kf.
103. ((extra or take home) adj10 (food* or ration or rations)).tw,kf.
104. Minerals/ and Dietary Supplements/
105. ((mineral or minerals) adj10 (diet* or nutrition*) adj3 supplement*).tw,kf.
106. sprinkles.tw,kf.
107. Pellagra/
108. ("italian leprosy" or "lombardy leprosy" or maidism or "niacin deficiency" or pellagra or pellagras or "pellagrous skin").tw,kf.
109. Nutrition Assessment/
110. ("dietary assessment*" or "dietary evaluation*" or "mininutrition* assessment*" or "nutrition* assessment*" or "nutrition* index" or "nutrition* indexes" or "nutrition* indices" or "nutrition* evaluation").tw,kf.
111. Nutrition Disorders/
112. "nutrition* surveillance".tw,kf.
113. Nutrition Surveys/
114. ("nutrition* survey*" or nhanes or "national health and nutrition examination survey" or "health survey" or "smart survey").tw,kf.
115. Nutritional Status/
116. ("nutrition* status" or "nutrition* state").tw,kf.
117. ("outpatient therapeutic" adj2 (care or program*)).tw,kf.
118. (("ready to use" adj3 ("supplementary food*" or "therapeutic food*")) or rusf or rutf).tw,kf.
119. "selective feeding".tw,kf.
120. ("stabilisation centre*" or "stabilisation center*" or "stabilization centre*" or "stabilization center*").tw,kf.
121. Starvation/
122. (famine or famines or starvation or starvations).tw,kf.
123. ("supplementary feeding*" or "complementary feeding*" or "complementary food" or "supplementary feeding*").tw,kf.
124. "targeted food distribution*".tw,kf.
125. "therapeutic feeding*".tw,kf.
126. "therapeutic food*".tw,kf.
127. Thiamine Deficiency/
128. ("thiamine deficienc*" or "aneurin deficienc*" or "avitaminosis b1" or "thiamin deficienc*" or "vitamin b 1 deficienc*" or "vitamin b1 deficienc*").tw,kf.
129. Beriberi/
130. (beriberi or "beri beri").tw,kf.
131. Vitamins/
132. (vitamin or vitamins).tw,kf.

133. Ascorbic Acid Deficiency/
134. ("ascorbic acid deficienc*" or "vitamin c deficienc*" or "avitaminosis c" or "hypovitaminosis c").tw,kf.
135. Scurvy/
136. (hypoascorbemia or hypoascorbemias or scorbutus or scurvies or scurvy or "barlow disease" or scorbut).tw,kf.
137. (transfer* adj10 (voucher or vouchers)).tw,kf.
138. Wasting Syndrome/
139. (wasting or wasted).tw,kf.
140. or/62-139

141. 61 and 140
142. 201804*.ed.
143. ("2018 04*" or "2018 05*" or "2018 06*" or "2018 07*" or "2018 08*" or "2018 09*" or "2018 10*" or "2018 11*" or "2018 12*" or 2019*).dt.
144. 141 not (142 or 143)
145. 141 not 142

Appendix 3. Characteristics of included studies

Author Name & Publication Year	Literature Type	Report Type	Country	Target Population	Displacement Status	Setting	Interventions	Delivery Platforms	Delivery Personnel	Delivery Sites
Aaby 1999 [1]	Indexed	RCT	Guinea-Bissau	9-24 months	Refugees, IDPs, Non displaced	Dispersed	GFD, Nutritional status assessment	NGO/UN agency	NGO/UN staff	Home
Abdulsalam 2016 [2]	Grey	Non-research	Palestine	Post-natal mothers	IDPs, Refugees	Camp, Dispersed	BF promotion	NGO/UN agency	CHWs	Home
Abu-Taleb 2015[3]	Grey	Non-research	Jordan	6-59 months, PLWs	Host, Refugees	Camp, Dispersed	Inpatient SAM treatment, MNS, Nutritional status assessment, Outpatient/Community-based TF, TSF	MOH, NGO/UN agency	CHWs, NGO/UN staff	Hospitals, Outpatient clinic, Mobile clinic
Alsamman 2015[4]	Grey	Non-research	Jordan	Children under 5, PLW, Care-givers	Host, Refugees	Camp, dispersed	BF promotion, Infant formula distribution, Nutrition education, Outpatient/Community-based TF, Inpatient SAM treatment	MOH, NGO/UN agency	CHWs, Doctors, IYCF counsellors, Midwives, Nurses	Hospital, Mother-baby friendly space caravans (safe haven)
Altmann 2017[5]	Indexed	Non-research	Yemen	Children under 5, All	IDPs, Non displaced	Dispersed	Inpatient SAM treatment	MOH, NGO/UN agency	CHWs	Hospital, Health centre
Barakat 2017[6]	Grey	Non-research	Lebanon	All	Non displaced, Refugees	Dispersed	GFD	NGO/UN agency	Volunteers	Community kitchens
Battistin 2016[7]	Grey	Non-randomized	Lebanon	All	Refugees	Camp, Dispersed	Cash/voucher distribution	NGO/UN agency	NGO/UN staff	Home

		controlled trial								
Berbari 2015[8]	Grey	Non-research	Lebanon	Children under5, PLW, All	Host, Refugees	Camp, dispersed	Inpatient SAM treatment, MNS, Nutritional status assessment, Nutrition education (IYCF), Outpatient /Community-based TF, TSF, Training	MOH, NGO/UN agency , Research	CHWs, Doctors, Nurses, NGO/UN staff	Hospitals , Clinics, Health centres, Home
Bile 2011[9]	Indexed	Non-research	Pakistan	Children under 5, Women	IDPs, Host	Camp, Dispersed	Nutritional status assessment, BF promotion, Inpatient SAM treatment, Outpatient/Community-based TF, Control of disease outbreak, disease prevention and management	MOH, NGO/UN agency	Health workers, Doctors, Nurses	Hospitals , Health centre, Mobile clinics
Bilukha 2011[10]	Indexed	Observational	Nepal	6-59 months	Refugees	Camp	GFD, TSF, MNS, Deworming, Management of common illness	NGO/UN agency	Health Workers	Health centre
Bilukha 2014[11]	Indexed	Non-research	Jordan	6-59 months, 15-49 year	Refugees, Host	Camp, Dispersed	Cash/voucher distribution, Nutritional status assessment	MOH, NGO/UN agency	Health workers	Health centre
Borrel 2001[12]	Indexed	Non-research	Macedonia	0-12 months, All	Refugees	Camp	GFD	NGO/UN agency	NGO/UN staff	NR
Callaghan 1995[13]	Indexed	Non-research	DRC	0-15 years	Refugees	Camp	GFD, Nutritional status assessment, Disease Management and Prevention	MOH, NGO/UN agency	Nutritionists, Nurses, NGO/UN staff	Health centre

Carrara 2017[14]	Indexed	Observational	Thailand	PW	Refugees	Camp	GFD, MNS	Research Organizatio n	NGO/UN staff	Clinic
CDC 1990[15]	Indexed	Non-research	Ethiopia	6-59 months	Refugees	Camp	BSF, MNS	NGO/UN agency	NR	Health centre
CDC 1991[16]	Indexed	Non-research	Guinea	6-59 months	Refugees, Hosts	Camp, Dispersed	GFD, Nutritional status assessment, Disease Management and Prevention	Military force, MOH, NGO/UN agency	NR	NR
CDC 1991[17]	Indexed	Non-research	Iraq	All	Refugees, IDPs	Camp	GFD	MOH, Defense system, NGO/UN agency	Military personnel, NGO/UN staff	NR
CDC 1995[18]	Indexed	Non-research	Sudan	Children under 5	Returning refugees	Dispersed	MNS, Nutrition education, Deworming	MOH, NGO/UN agency	Health workers, Volunteer s	Health centre
Charchuk 2015[19]	Indexed	Observational	South Sudan	5-19 years	IDPs, Returning refugees	Dispersed	Nutritional status assessment	NGO/UN agency	NGO/UN staff	Schools
Chaudhry 2018[20]	Indexed	Observational	West Bank and Gaza	6-59 months	Refugees	Camp, Dispersed	Food fortification, MNS	MOH, NGO/UN agency	Health workers, NGO/UN staff	Clinic
Cheung 2003[21]	Indexed	Non-research	Afghanist an	Children under 5, All	Refugees, Non displaced	Dispersed	BSF, MNS, Nutrition education	MOH, NGO/UN agency	CHWs	Distributi on points
Chinjekure 2018[22]	Grey	Non-research	Afghanist an	Children under 5, PLW	NR	NR	Nutritional status assessment	MOH, NGO/UN agency	CHWs, NGO/UN staff, Health workers	Hospitals , Clinics, health posts

Ciglenecki 2011[23]	Indexed	Observational	Cameroon	All	Refugees, Host	Camp, Dispersed	Nutrition education, Outpatient/Community-based TF	NGO/UN agency	CHWs, Doctors, NGO/UN staff	Mobile clinic
Collins 1998[24]	Indexed	Observational	Somalia	All	NR	Dispersed	Outpatient/Community-based TF	MOH, NGO/UN agency	Doctors, Nurses , NGO/UN staff	TFCs
Colombatti 2008[25]	Indexed	Observational	Guinea-Bissau	1-17 years	Non displaced	Dispersed	Inpatient SAM treatment, Outpatient/Community-based TF, MNS, Nutrition education, Deworming	MOH, NGO/UN agency	Doctors, Nurses, Volunteers	Hospital
Daniel 2016[26]	Grey	Non-research	Sudan	Children under5	IDPs, Non displaced	Dispersed	Inpatient SAM treatment, Outpatient /Community-based TF	MOH, NGO/UN agency, Research	Health workers, Nutritionist	Clinics, SCs
Darjani 2014[27]	Grey	Non-research	Lebanon	Children under 2, PLW	Host, Refugees	NR	BF promotion, Complementary feeding promotion,Infant formula provision, Nutrition education (Complementary feeding, IYCF during illness, infant formula use), Training	MOH, NGO/UN agency	Health workers, NGO/UN staff, Nutritionist	Hospitals , Clinics, Health centres
Davidson 2015[28]	Grey	Non-research	Lebanon	Children under5, All	Refugees	Dispersed	Nutritional status assessment	MOH, NGO/UN agency	Doctor, Nurse, CHW	Mobile clinics
Desie 2017[29]	Grey	Non-research	Somalia	6-59 months, PLW	IDPs, Non displaced	Camp, Dispersed	Nutritional status assessment, BF promotion,	MOH, NGO/UN agency	Health workers	Mobile clinics

							Outpatient/Community-based TF			
Doocy 2011[30]	Indexed	Observational	Jordan<br>Syria	All	Refugees	Camp, Dispersed	GFD, Cash/voucher distribution	NGO/UN agency	NGO/UN staff	Home
Doocy 2017[31]	Indexed	Observational	Syria	All	IDPs	Dispersed	GFD , Cash/voucher distribution	NGO/UN agency	NGO/UN staff	Distribution points
Dozio 2016[32]	Indexed	Non-research	Central African Republic	PLW	IDPs	Camp	Cash/voucher distribution, Nutrition education	NGO/UN agency	NGO/UN staff	NGO clinic
Duckett 1996[33]	Indexed	Non-research	Rwanda	All Women, PW	Refugees	Camp	GFD, MNS, Disease Prevention and Management	NGO/UN agency	NGO/UN staff	NGO clinic
Dureab 2016[34]	Grey	Non-research	Yemen	Children under 5	Non displaced	NR	Inpatient SAM treatment, Outpatient/Community-based TF, TSF, Nutrition education (Complementary feeding, IYCF during illness, infant formula use), Training	MOH, NGO/UN agency	Doctors, Nurses, Health workers, NGO/UN staff, National and International WHO nutrition experts	Hospital, TFCs
Dzumhur 1995[35]	Indexed	Observational	Bosnia and Herzegovina	1-14 years	Non displaced	Dispersed	Therapeutic feeding	MOH, NGO/UN agency	Health workers	Clinic, Health centre

Egendal 2015[36]	Grey	Non-research	Syria	6-12 years	IDPs	Dispersed	GFD	MOE, NGO/UN agency	NGO/UN staff	Schools
El-Huni 2015[37]	Grey	Non-research	Lebanon	All	Refugees	Dispersed	Food vouchers/cash provision	MOH, NGO/UN agency , Research	NGO/UN staff	Electroni c/print
Eltom 2001[38]	Indexed	Non-research	Afghanist an	Children under 5, All	IDPs	Camp, Dispersed	GFD, Outpatient/Communi ty-based TF, Training, Disease Prevention and Management	NGO/UN agency	NGO/UN staff	SFC
Fander 2014[39]	Indexed	Observational	Jordan	0-6 months, their mothers	Refugees	Dispersed	BF promotion, Complementary feeding promotion	NGO/UN agency	NR	Home
Fander 2015[40]	Grey	Non-research	Jordan	Children under 5, PLW	Refugees	Camp, Dispersed	BF promotion, Nutritional status assessment, Nutrition education	MOH, NGO/UN agency	Health workers	Mobile clinics
Farah 2014[41]	Grey	Non-research	Somalia	5-15 years	Non displaced	Dispersed	BSF, GFD	MOE, NGO/UN agency	NGO/UN staff	Schools
Feldman 2013[42]	Grey	Non-research	Colombia	All	IDPs, Refugees	Camp	GFD	NGO/UN agency	NGO/UN staff	Market
Fournier 1999[43]	Indexed	Non-research	Burundi	Children under 5, All	IDPs	Camp	Outpatient/Communi ty-based TF, Training	MOH, NGO/UN agency	NGO/UN staff	TFCs, SFCs
Giordano 2017[44]	Grey	Non-research	Jordan	All	Refugees	Camp	Food vouchers/cash provision	NGO/UN agency	NGO/UN staff	Market
Hoetjes 2015[45]	Grey	Non-research	Syria	Children under 5	IDPs	Dispersed	Nutritional status assessment, Outpatient/Communi ty-based TF	MOH, NGO/UN agency	Health workers, NGO/UN staff	TFCs, Mobile clinics

Inglis 2014[46]	Grey	Non-research	Turkey	All	Refugees	Camp	GFD, Cash/voucher distribution	MOH, NGO/UN agency	NGO/UN staff, MOH staff	Market
IRC 2018[47]	Grey	Observational	South Sudan	Children under 5, and their mothers	NR	NR	BF promotion, Outpatient/Community-based TF, Nutrition education, Training, Inpatient SAM treatment	NGO/UN agency	NGO/UN staff, Community based distributors (CBDs)	Clinic, Home
Jayatissa 2012[48]	Indexed	Observational	Sri Lanka	Children under 5	IDPs	Camp	BF promotion (with infant formula feeding where indicated, under supervision), Nutritional status assessment, Inpatient SAM treatment, Outpatient/Community-based TF, TSF, BSF, MNS, Deworming, Training	MOH, NGO/UN agency	Health workers, Doctors, MOH staff, Volunteers	Hospital, Mobile clinic, Health centre
Karagueuzian 2017[49]	Grey	Non-research	Lebanon	5-14 years	Host, Refugees	Dispersed	BSF, Nutrition education	MOE,NGO/UN agency	NGO/UN staff	Schools
Karimova 2014[50]	Grey	Non-research	Lebanon	Children under5, PLW	Host, Refugees	Camp, Dispersed	Inpatient SAM treatment, MNS, Nutritional status assessment, Outpatient/Community-based TF, ORS treatment of diarrhoea	MOH, NGO/UN agency	Doctors, Nurses,Nutritionist, CHWs , NGO/UN staff	Hospital Mobile clinics, Home
Kassim 2012[51]	Indexed	RCT	Kenya	PW	Refugees	Camp	MNS	NGO/UN agency, Research	NGO/UN staff	NGO clinic

Khatib 2010[52]	Indexed	Observational	Jordan	6-59 months, Women	Refugees	Camp	Nutritional status assessment	NGO/UN agency, Research	NGO/UN staff	Home
Khudari 2015[53]	Grey	Non-research	Syria	Children under 5, Post-natal mothers, All	Host, IDPs	Camp, Dispersed	Nutritional status assessment, BF promotion, MNS, Inpatient SAM treatment, Outpatient/community-based TF, TSF, BSF, Nutrition education (BF/IYCF), Training	Healthcare system, NGO/UN agency	CHWs, NGO/UN staff	Hospital, Clinics
Kumar 2003[54]	Indexed	Observational	India	6-59 months, 5-14 years	IDPs	Camp	MNS, Disease Prevention and Management	MOH, NGO/UN agency	Doctors, MOH health staff	Health post
Laker 2016[55]	Grey	Non-research	South Sudan	Children under5, PLW	IDPs	Dispersed	Inpatient SAM treatment, Nutritional status assessment, Outpatient/ community-based therapeutic feeding, TSF	MOH, NGO/UN agency	community nutrition volunteers , MOH staff,	Clinic, SCs
Leidman 2017[56]	Indexed	Non-research	Nigeria	6-59 months	IDPs, Refugees, Non displaced	Camp, Dispersed	Nutritional status assessment, Deworming	MOH, NGO/UN agency	NR	Home
Leonardi 2013[57]	Grey	Non-research	Mali	Children under5	IDPs, Refugees	Dispersed	Outpatient/Community-based TF	MOH, NGO/UN agency	Health workers	Health centre
Leus 1993[58]	Indexed	Non-research	Bosnia and Herzegovina	All	IDPs, Non displaced	Camp, Dispersed	GFD	NGO/UN agency	NGO/UN staff	Mobile clinics

Lopriore 2004[59]	Indexed	RCT	Algeria	3-6 years	Refugees	Camp	GFD, MNS	MOH, Research	Research staff, Health workers	SFCs
Magoni 2008[60]	Indexed	Observational	West Bank and Gaza	6-59 months, PW	IDPs, Non displaced	Dispersed	MNS, Nutrition education, TSF	NGO/UN agency, MOH	Health workers	Clinic, Home
Mahomed 2012[61]	Indexed	Non-research	Somalia	All	IDPs	Camp	GFD	NGO/UN agency	NGO/UN staff	Feeding centres
Malfait 1991[62]	Indexed	Non-research	Malawi	All	Refugees	Camp, Dispersed	GFD, MNS	MOH, NGO/UN agency	NGO/UN staff	NR
McGready 2001[63]	Indexed	Observational	Thailand	PLW	Refugees	Camp	GFD, MNS	NGO/UN agency, Research Organization	NR	NR
Morris 2012[64]	Indexed	Observational	Uganda	6-30 months and their mothers	IDPs	Camp	Inpatient SAM treatment, Outpatient/Community-based TF	MOH, NGO/UN agency	Health workers	Feeding centres, Home
Morseth 2017[65]	Indexed	Observational	Algeria	All >18 years	Refugees	Camp	GFD	NGO/UN agency	NGO/UN staff	NR
Murphy 1996[66]	Indexed	Cluster-RCT	Pakistan	6-59 months	Refugees	Camp	Nutrition education on feeding during diarrhea; ORS, Disease Prevention and Management	MOH, NGO/UN agency	CHWs, Doctors	MOH clinic, Home
Murphy 2017[67]	Grey	Non-research	Ethiopia	Children under5, PLW	Refugees	Camp	Inpatient SAM treatment, Nutritional status assessment, Outpatient/Community-based TF, TSF, Nutrition education	MOH, NGO/UN agency	Nurses, NGO/UN staff, Health workers, Counsellors	Clinic, SCs

Ndemwa 2011[68]	Indexed	Observational	Kenya	6-59 months, Women 18-49y ears	Refugees	Camp	GFD, MNS	MOH, NGO/UN agency	CHWs, Nutritionists	Distribution point
Ndungu 2017[69]	Grey	Non-research	South Sudan	Children under 5, All aged 15-19y, PLW	IDPs	NR	BF promotion, Complementary feeding promotion, Nutrition education	NGO/UN agency	NGO/UN staff, Volunteers	Home
Nielsen 2004[70]	Indexed	Observational	Guinea-Bissau	6-59 months	IDPs, Non displaced	Camp	Breastfeeding promotion, MNS, TSF	MOH, Research	Health worker	Health centre, Home
Nielsen 2005[71]	Indexed	Observational	Guinea-Bissau	6-59 months	Returning refugees	Camp	Breastfeeding promotion, MNS, TSF	MOH, Research	Health worker	Health centre, Home
O'Mahony 2013[72]	Grey	Non-research	Kenya	All	IDPs	Camp, Dispersed	Food vouchers/cash provision, Training	NGO/UN agency	Health workers, NGO/UN staff	Health facilities, Electronic/print
Renzaho 2003[73]	Indexed	Observational	DRC	6-59 months	Refugees	Camp	Inpatient SAM treatment, Outpatient/Community-based TF, Immunization	MOH, NGO/UN agency	Nutritionists, CHWs	Hospital, TFCs
Rutta 2008[74]	Indexed	Observational	Tanzania	PW	Refugees	Camp	Infant feeding support, Nutrition education , HIV treatment	MOH, NGO/UN agency	Health worker	TFC, Home
Sallam 2018[75]	Grey	Non-research	Yemen	Children under5, PW	Non displaced	N/A	Deworming, Nutritional status assessment (and referral for treatment), MNS, Nutrition education (IYCF, WASH)	MOH, NGO/UN agency	Community health volunteers, NGO/UN staff	Home

Salse 2013[76]	Grey	RCT	Uganda	1-59 months	NR	NR	Nutritional status assessment, Outpatient/community-based TF, MNS, Therapeutic treatment with ORS and Zinc	MOH, NGO/UN agency	Coordinator, study dispenser	Hospitals, Clinics
Sami 2017[77]	Indexed	Observational	South Sudan	All >18 years	IDPs, Refugees	Camp	Nutrition education	NGO/UN agency	NGO/UN staff	Hospital
Schramm 2013[78]	Indexed	Observational	Uganda	All >15 years	IDPs	Camp	GFD, Nutritional status assessment, Training	NGO/UN agency	NGO/UN staff	NR
Seal 2008[79]	Indexed	Observational	Zambia	6-59 months, 10-19 years, Women 20-49 years	Refugees	Camp	Food fortification, Training	MOH, NGO/UN agency	NGO/UN staff, Nutritionists	Distribution point
Sebuliba 2015[80]	Grey	Non-research	Jordan	6-23 months, All Girls, PLW	Host, Refugees	Camp, Dispersed	Nutritional status assessment, TSF, SAM/MAM treatment	MOH, NGO/UN agency	NGO/UN staff	Clinics
Seguin 2014[81]	Grey	Non-research	Lebanon	0-24 months, PLW, caregivers	Refugees	Camp	BF promotion Complementary feeding promotion, Nutrition education	NGO/UN agency	NGO/UN staff	ACF tents ("Safe Havens")
Stuetz 2016[82]	Indexed	Observational	Thailand	PW	Refugees	Camp	GFD, MNS	Research Organization	NGO/UN staff	Clinic
Talley 2009[83]	Indexed	Observational	Tanzania	Children under 5 and their mothers	Refugees	Camp	Distribution of stainless steel (Fe alloy) cooking pots	MOH, NGO/UN agency	NGO/UN staff	Distribution point

Tappis 2012[84]	Indexed	Observational	Kenya	6-59 months	Refugees	Camp	Inpatient SAM treatment, Outpatient/community-based TF, Nutritional status assessment, TSF, Disease management and prevention	MOH, NGO/UN agency	CHWs	SFC
			Tanzania							
Tchamba 2017[85]	Grey	Non-research	DRC	6-59 months, PLW	IDPs	Dispersed	Inpatient SAM treatment, Nutritional status assessment, Nutrition education, Outpatient /Community-based TF,	MOH, NGO/UN agency	CHWs, NGO/UN staff	Hospital, Health centre, Health post, Mobile clinics,
Toole 1992[86]	Indexed	Observational	Ethiopia	6-59 months	Refugees	Camp	BSF, MNS	MOH, NGO/UN agency	NGO/UN staff	Hospital
Van Den Briel 2007[87]	Indexed	Observational	Angola	Children under 5, All	IDPs,	Dispersed	Food fortification, GFD	MOH, NGO/UN agency	NGO/UN staff	Home
			Zambia		Refugees	Camp				TFCs
			Afghanistan		IDPs	Dispersed				Home
Van Der Merwe 2014[88]	Grey	Non-research	South Sudan	All	Refugees	Camp	GFD, Cash/voucher distribution	NGO/UN agency	NGO/UN staff	Market
Vautier 1999[89]	Indexed	Non-research	Burundi	6-59 months	Returning refugees	Dispersed	TSF, Disease Prevention and Management	MOH, NGO/UN agency	Health workers	SFCs
			DRC		Refugees	Camp				
			Liberia		NR	Dispersed				

Watson 1995[90]	Indexed	Observational	Bosnia and Herzegov ina	All	IDPs, Refugees, Non displaced	Camp, Dispersed	GFD, Nutritional status assessment	NGO/UN agency	NR	NR
Yunusu 2016[91]	Grey	Non-research	South Sudan	All	IDPs	Dispersed	GFD, Cash/voucher distribution	NGO/UN agency	NGO/UN staff	Market

References

1. Aaby P, Gomes J, Fernandes M, et al. Nutritional status and mortality of refugee and resident children in a non-camp setting during conflict: follow up study in Guinea-Bissau. *British Medical Journal* 1999;319
2. Abdulsalam W, Masri L. Ex-Post Evaluation of UNICEF Humanitarian Action for Children 2014-2015 in the State of Palestine. 2016
3. Abu-Taleb R. Experiences of emergency nutrition programming in Jordan. *Field Exchange* 2015(48):93.
4. Alsamman S. Managing infant and young child feeding in refugee camps in Jordan. *Field Exchange* 2014(48):85.
5. Altmann M, Suarez-Bustamante M, Soulier C, et al. First Wave of the 2016-17 Cholera Outbreak in Hodeidah City, Yemen - ACF Experience and Lessons Learned. *PLoS Curr* 2017;9 doi: 10.1371/currents.outbreaks.5c338264469fa046ef013e48a71fb1c5
6. Barakat J. Community kitchens in Lebanon: Cooking together for health. *Nutrition Exchange* 2017(8):9.
7. Battistin F. Impact evaluation of the multipurpose cash assistance programme. 2016
8. Berbari L, Ousta D, Asfahani F. Institutionalising acute malnutrition treatment in Lebanon. *Field Exchange* 2014(48)
9. Bile K, Hafeez A, Kazi G, et al. Protecting the right to health of internally displaced mothers and children: the imperative of inter-cluster coordination for translating best practices into effective participatory action. *Eastern Mediterranean Health Journal* 2011;17(12):981-89.
10. Bilukha O, Howard C, Wilkinson C, et al. Effects of multimicronutrient home fortification on anemia and growth in Bhutanese refugee children. *Food and Nutrition Bulletin* 2011;32(3):264-76.
11. Bilukha O, Jayasekaran D, Burton A, et al. Nutritional Status of Women and Child Refugees from Syria — Jordan, April–May 2014. *Morbidity and Mortality Weekly Report* 2014;63(29):638-39.
12. Borrel A, McGrath M, Hormann E, et al. From policy to practice: Challenges in infant feeding in emergencies during the Balkan crisis. *Disasters* 2001;25(2):149-63.
13. Callaghan M, Immerman B. PHS Mission to Goma, Zaire. *Public Health Reports* 1995;110(1):95-99.
14. Carrara VI, Stuetz W, Lee SJ, et al. Longer exposure to a new refugee food ration is associated with reduced prevalence of small for gestational age: results from 2 cross-sectional surveys on the Thailand-Myanmar border. *Am J Clin Nutr* 2017;105(6):1382-90. doi: 10.3945/ajcn.116.148262
15. Centre for Disease Control. International notes update: Health and nutritional profile of refugees -- Ethiopia, 1989- 1990. *Morbidity and Mortality Weekly Report* 1990;39(40)
16. Centre for Disease Control. Health and nutritional status of Liberian refugee children, 1990. *Weekly Epidemiological Record* 1991 (A)(16)
17. Centre for Disease Control. Public health consequences of acute displacement of Iraqi citizens - March-May 1991. *Journal of the American Medical Association* 1991 (B);266(5):633-34.
18. Centre for Disease Control. Implementation of health initiatives during a cease-fire -- Sudan, 1995. *Morbidity and Mortality Weekly Report* 1995;44(23):433-36.
19. Charchuk R, Houston S, Hawkes MT. Elevated prevalence of malnutrition and malaria among school-aged children and adolescents in war-ravaged South Sudan. *Pathog Glob Health* 2015;109(8):395-400. doi: 10.1080/20477724.2015.1126033
20. Chaudhry AB, Hajat S, Rizkallah N, et al. Risk factors for vitamin A and D deficiencies among children under-five in the state of Palestine. *Confl Health* 2018;12:13. doi: 10.1186/s13031-018-0148-y
21. Cheung E, Mutahar R, Assefa F, et al. An epidemic of scurvy in Afghanistan: Assessment and response. *Food and Nutrition Bulletin* 2003;24(3):247-55.
22. Chinjekure A, Shams DM, Qureshi DA, et al. Screening for maternal and child malnutrition using sentinel-based national nutrition surveillance in Afghanistan. *Field Exchange* 2018(58)
23. Ciglenečki I, Eyema R, Kabanda C, et al. Konzo outbreak among refugees from Central African Republic in Eastern region, Cameroon. *Food and Chemical Toxicology* 2011;49(3):579-82. doi: 10.1016/j.fct.2010.05.081
24. Collins S, Myatt M, Golden B. Dietary treatment of severe malnutrition in adults. *American Journal of Clinical Nutrition* 1998;68:193-99.

25. Colombatti R, Coin A, Bestagini P, et al. A short-term intervention for the treatment of severe malnutrition in a post-conflict country: results of a survey in Guinea Bissau. *Public Health Nutr* 2008;11(12):1357-64. doi: 10.1017/S1368980008003297
26. Daniel T, Mekkawi T, Garelnabi H, et al. Scaling up CMAM in protracted emergencies and low resource settings: experiences from Sudan. *Field Exchange* 2016(55):74.
27. Darjani P, Berbari L. Infant and young child feeding support in Lebanon:\ strengthening the national system. *Field Exchange* 2014(48):20.
28. Davidson J, Bethke C. Integrating community-based nutrition awareness into the Syrian refugee response in Lebanon. *Field Exchange* 2015(48):29.
29. Desie S. Somalia Nutrition Cluster: integrated famine prevention package. *Field Exchange* 2017:53-55.
30. Doocy S, Sirois A, Anderson J, et al. Food security and humanitarian assistance among displaced Iraqi populations in Jordan and Syria. *Soc Sci Med* 2011;72(2):273-82. doi: 10.1016/j.socscimed.2010.10.023
31. Doocy S, Tappis H, Lyles E, et al. Emergency Food Assistance in Northern Syria: An Evaluation of Transfer Programs in Idleb Governorate. *Food Nutr Bull* 2017;38(2):240-59. doi: 10.1177/0379572117700755
32. Dozio E, Peyre L, Morel S, et al. Integrated psychosocial and food security approach in an emergency context: Central African Republic. *Intervention* 2016;14(3):257-71.
33. Duckett J. Guidelines for dietary supplementation of pregnant women in a Rwandan refugee camp. *Journal of the Royal Army Medical Corps* 1996;142(1):13-14.
34. Dureab F, Jawaldeh DA, Abbas DL. Building capacity in inpatient treatment of severe acute malnutrition in Yemen. *Field Exchange* 2016(55):87.
35. Dzumhur Z, Zec S, Buljina A, et al. Therapeutic feeding in Sarajevo during the war. . *European journal of clinical nutrition* 1995;49(Suppl 2):S40-2.
36. Egendal R, Badejo A. WFP's emergency programme in Syria. *Field Exchange* 2015(48)
37. El-Huni E. WFP e-voucher programme in Lebanon. *Field Exchange* 2015(48):36.
38. Eltom AA. Internally displaced people—refugees in their own country. *The Lancet* 2001;358(9292):1544-45. doi: 10.1016/s0140-6736(01)06589-8
39. Fander G, Beck N, Johan H, et al. Extremely low exclusive breast feeding (ebf) rate among the Syrian refugee communities in Jordan. *Archives of Diseasese in Childhood* 2014;99(Supplement 2):A226.
40. Fänder G, Frega M. Responding to nutrition gaps in Jordan in the Syrian Refugee Crisis: Infant and Young Child Feeding education and malnutrition treatment. *Field Exchange* 2014(48):82.
41. Farah A. School feeding: experiences from Somalia. *Nutrition Exchange* 2014(4):17.
42. Feldman S, Freccero J, Seelinger K. Safe haven: Sheltering displaced persons from sexual and gender-based violence case study: Colombia. 2013
43. Fournier A, Mason F, Peacocke B, et al. The management of severe malnutrition in Burundi: An NGO's perspective of the practical constraints to effective emergency and medium-term programmes. *Disasters* 1999;23(2):343-49.
44. Giordano, Dunlop K, Gabay T, et al. Evaluation synthesis of UNHCR's cash based interventions in Jordan. 2017
45. Hoetjes M, Rhymer W, Matasci-Phelippeau L, et al. Emerging cases of malnutrition amongst IDPs in Tal Abyad district, Syria. *Field Exchange* 2015(48):133.
46. Inglis K, Vargas J. Experiences of the e-Food card programme in the Turkish refugee camps. *Field Exchange* 2014
47. International Rescue Committee. Enabling treatment of severe acute malnutrition in the community: Study of a simplified algorithm and tools in South Sudan. 2018
48. Jayatissa R, Bekele A, Kethiswaran A, et al. Community-based management of severe and moderate acute malnutrition during emergencies in Sri Lanka: Challenges of implementation. *Food and Nutrition Bulletin* 2012;33(4):251-60.
49. Karagueuzian N. Healthy snacks and nutrition education: School feeding in Lebanon's public schools. *Nutrition Exchange* 2017(8):10.
50. Karimova J, Hammoud J. Relief International nutrition and health programme in Lebanon. *Field Exchange* 2014(48):27.

51. Kassim IAR, Ruth LJ, Creeke PI, et al. Excessive iodine intake during pregnancy in Somali refugees. *Maternal & Child Nutrition* 2012;8(1):49-56. doi: 10.1111/j.1740-8709.2010.00259.x
52. Khatib I, Samrah S, Zghol F. Nutritional interventions in refugee camps on Jordan's eastern border: assessment of status of vulnerable groups. *Eastern Mediterranean Health Journal* 2010;16(2):187-93.
53. Khudari H, Bozo M, Hoff E. WHO response to malnutrition in Syria: a focus on surveillance, case detection and clinical management. *Field Exchange* 2015(48):118.
54. Kumar V, Chaudhury L, Rathore R, et al. An epidemiological analysis of outbreak of measles in a medical relief camp. *Health and Population-Perspectives and Issues* 2003;26(4):135-40.
55. Laker M, Toose J. Nutrition programming in conflict settings: Lessons from South Sudan. *Field Exchange* 2016(53):2.
56. Leidman E, Tromble E, Yermina A, et al. Acute malnutrition among children, mortality, and humanitarian interventions in conflict-affected regions — Nigeria, October 2016–March 2017. *Morbidity and Mortality Weekly Report* 2017;66(48):1332-35.
57. Leonardi E, Arqués R. Real time evaluation of unicef's response to the Mali crisis final report. 2013
58. Leus X. Humanitarian assistance: Technical assessment and public health support for coordinated relief in the former Yugoslavia. *World Health Statistics Quarterly* 1993;46(3):199-203.
59. Lopriore C, Guidoum Y, Briend A, et al. Spread fortified with vitamins and minerals induces catch-up growth and eradicates severe anemia in stunted refugee children aged 3–6 y. *American Journal of Clinical Nutrition* 2004;80:973-81.
60. Magoni M, Jaber M, Piera R. Fighting anaemia and malnutrition in Hebron (Palestine): impact evaluation of a humanitarian project. *Acta Trop* 2008;105(3):242-8. doi: 10.1016/j.actatropica.2007.11.007
61. Mahomed Z, Moolla M, Motara F, et al. A Somalia mission experience. *South African Medical Journal* 2012;102(8):659. doi: 10.7196/samj.5970
62. Malfait P, Moren A, Malenga G, et al. Outbreak of pellagra among Mozambican refugees - Malawi, 1990. *Morbidity and Mortality Weekly Report* 1991;40(13):209-13.
63. McGready R, Simpson J, Cho T, et al. Postpartum thiamine deficiency in a Karen displaced population. *American Journal of Clinical Nutrition* 2001;74:808-13.
64. Morris J, Jones L, Berrino A, et al. Does combining infant stimulation with emergency feeding improve psychosocial outcomes for displaced mothers and babies? A controlled evaluation from northern Uganda. *Am J Orthopsychiatry* 2012;82(3):349-57. doi: 10.1111/j.1939-0025.2012.01168.x
65. Morseth MS, Grewal NK, Kaasa IS, et al. Dietary diversity is related to socioeconomic status among adult Saharawi refugees living in Algeria. *BMC Public Health* 2017;17(1):621. doi: 10.1186/s12889-017-4527-x
66. Murphy H, Bari A, Molla A, et al. A field trial of wheat-based oral rehydration solution among Afghan refugee children. *Acta Pediatrica* 1996;85
67. Murphy M, Kassahun Abebe, O'Mahony S, et al. Management of acute malnutrition in infants less than six months in a South Sudanese refugee population in Ethiopia. *Field Exchange* 2017(55):70.
68. Ndemwa P, Klotz C, Mwaniki D, et al. Relationship of the availability of micronutrient powder with iron status and hemoglobin among women and children in the Kakuma Refugee Camp, Kenya. *Food and Nutrition Bulletin* 2011;32(3):286-91.
69. Ndungu P, Tanaka J. Using care groups in emergencies in South Sudan. *Field Exchange* 2017(54):95-97.
70. Nielsen J, Valentiner-Branth P, Martins C, et al. Malnourished children and supplementary feeding during the war emergency in Guinea-Bissau in 1998–19991. *American Journal of Clinical Nutrition* 2004;80:1036-42.
71. Nielsen J, Benn CS, Bale C, et al. Vitamin A supplementation during war-emergency in Guinea-Bissau 1998-1999. *Acta Trop* 2005;93(3):275-82. doi: 10.1016/j.actatropica.2004.11.007
72. O'Mahony A, MacAuslan I. Evaluation of post 2007 election violence recovery programme in Kenya. *Field Exchange* 2013(46: Special focus on urban food security & nutrition):65.
73. Renzaho A, Renzaho C. In the shadow of the volcanoes: the impact of intervention on the nutrition and health status of Rwandan refugee children in Zaire two years on fromthe exodus. *Nutrition & Dietetics* 2003;60(2):85-91.
74. Rutta E, Gongo R, Mwansasu A, et al. Prevention of mother-to-child transmission of HIV in a refugee camp setting in Tanzania. *Glob Public Health* 2008;3(1):62-76. doi: 10.1080/17441690601111924

75. Sallam DF, Albably K, Zvandaziva C, et al. Community engagement through local leadership: Increasing access to nutrition services in a conflict setting in Yemen Increasing access to nutrition services in a conflict setting in Yemen. *Nutrition Exchange* 2018(9):10.
76. Salse N, Salse N, Swarthout T, et al. Effectiveness of nutritional supplementation (readyto- use therapeutic food and multi micronutrient) in preventing malnutrition in children 6-59 months with infection (malaria, pneumonia, diarrhoea) in Uganda. 2013
77. Sami S, Kerber K, Tomczyk B, et al. "You have to take action": changing knowledge and attitudes towards newborn care practices during crisis in South Sudan. *Reprod Health Matters* 2017;25(51):124-39. doi: 10.1080/09688080.2017.1405677
78. Schramm S. Nutritional status among adults in a post-conflict area, northern Uganda: are humanitarian assistance programmes creating disparities in health? *Eur J Epidemiol* 2013;28 Suppl 1:S182. doi: 10.1007/s10654-013-9820-0
79. Seal A, Kafwembe E, Kassim IA, et al. Maize meal fortification is associated with improved vitamin A and iron status in adolescents and reduced childhood anaemia in a food aid-dependent refugee population. *Public Health Nutr* 2008;11(7):720-8. doi: 10.1017/S1368980007001486
80. Sebuliba H, El-Zubi F. Meeting Syrian refugee children and women nutritional needs in Jordan. *Field Exchange* 2015(48):74.
81. Seguin J. Challenges of IYCF and psychosocial support in Lebanon. *Field Exchange* 2014(48):24.
82. Stuetz W, Carrara VI, Mc Gready R, et al. Impact of Food Rations and Supplements on Micronutrient Status by Trimester of Pregnancy: Cross-Sectional Studies in the Maela Refugee Camp in Thailand. *Nutrients* 2016;8(2):66. doi: 10.3390/nu8020066
83. Talley L, Woodruff BA, Seal A, et al. Evaluation of the effectiveness of stainless steel cooking pots in reducing iron-deficiency anaemia in food aid-dependent populations. *Public Health Nutr* 2010;13(1):107-15. doi: 10.1017/S1368980009005254
84. Tappis H, Doocy S, Haskew C, et al. United Nations High Commissioner for Refugees feeding program performance in Kenya and Tanzania: A retrospective analysis of routine Health Information System data. *Food and Nutrition Bulletin* 2012;33(2):150-60.
85. Tchamba A. Alert and rapid response to nutritional crisis in DRC. *Field Exchange* 2017(54):3.
86. Toole M, Bhatia R. A case study of Somali refugees in Hartisheik a camp, eastern ethiopia: Health and nutrition profile, July 1988-June 1990. *Journal of Refugee Studies* 1992;5(3/4):313-26.
87. van der Briel T, Cheung E, Zewari J, et al. Fortifying food in the field to boost nutrition: Case studies from Afghanistan, Angola, and Zambia. *Food and Nutrition Bulletin* 2007;28(3):353-64.
88. Van Der Merwe R. Impact of milling vouchers on household food security in South Sudan. *Field Exchange* 2014(47)
89. Vautier F, Hilderbrand K, Dedeurwaeder M, et al. Dry supplementary feeding programmes: an effective short- term strategy in food crisis situations. *Tropical Medicine and International Health* 1999;4(12):875-79.
90. Watson F, Kulenovic I, Vespa J. Nutritional status and food securtiy: Winter nutrition monitoring in Sarajevo1993-1994. *European Journal of Clinical Nutrition* 1995;49(Supplement 2):S23-S32.
91. Yunusu E, Markhan M. Cash-based programming to address hunger in conflict-affected South Sudan: A case study. 2016
